# High‐density MRI coil arrays with integrated field monitoring systems for human connectome mapping

**DOI:** 10.1002/mrm.30606

**Published:** 2025-06-18

**Authors:** Mirsad Mahmutovic, Manisha Shrestha, Gabriel Ramos‐Llordén, Dongsuk Sung, Luke J. Edwards, Ying Chu, Paul I. Dubovan, Alina Müller, Sam‐Luca J.D. Hansen, Anpreet Ghotra, Kerrin J. Pine, Roland Müller, Nikolaus Weiskopf, Lawrence L. Wald, Choukri Mekkaoui, Harald E. Möller, Susie Y. Huang, Boris Keil

**Affiliations:** ^1^ Institute of Medical Physics and Radiation Protection TH‐Mittelhessen University of Applied Sciences Giessen Hesse Germany; ^2^ Athinoula A. Martinos Center for Biomedical Imaging, Department of Radiology Massachusetts General Hospital, Harvard Medical School Boston Massachusetts USA; ^3^ Department of Neurophysics Max Planck Institute for Human Cognitive and Brain Sciences Leipzig Saxony Germany; ^4^ Nuclear Magnetic Resonance Methods and Development Group Max Planck Institute for Human Cognitive and Brain Sciences Leipzig Saxony Germany; ^5^ Felix Bloch Institute for Solid State Physics, Faculty of Physics and Earth Sciences Leipzig University Leipzig Saxony Germany; ^6^ Wellcome Centre for Human Neuroimaging, Institute of Neurology University College London London UK; ^7^ Harvard‐MIT Division of Health Sciences and Technology Massachusetts Institute of Technology Cambridge Massachusetts USA; ^8^ Department of Diagnostic and Interventional Radiology, University Hospital Marburg Philipps University of Marburg Marburg Hesse Germany; ^9^ LOEWE Research Cluster for Advanced Medical Physics in Imaging and Therapy (ADMIT) TH‐Mittelhessen University of Applied Sciences Giessen Hesse Germany

**Keywords:** diffusion MRI, field monitoring, human connectome project, MRI radiofrequency coil, phased array coil

## Abstract

**Purpose:**

To develop and test two high‐density MRI coil arrays with integrated field monitoring systems for enhanced diffusion imaging with strong diffusion‐sensitizing gradients.

**Methods:**

Two multichannel head coils were constructed for first‐ and second‐generation 3T Connectome MRI scanners, incorporating 64 and 72 receive channels, respectively. The array coils were evaluated using RF bench‐level metrics, including quality factor, tuning, matching, and coupling measurements. Imaging performance was comprehensively assessed through metrics such as SNR, $B_{1}^{+}$ efficiency, and inter‐channel noise correlations, and compared with and without field camera integration. Parallel imaging capability was evaluated using geometry (g)‐factors. The field camera performance was characterized by quantifying phase errors and field probe FID lifetimes. In vivo DWI acquisitions with high b‐values were performed to evaluate the system's ability to correct higher‐order field perturbations.

**Results:**

The developed arrays demonstrated up to 1.4‐fold higher SNR and superior g‐factor performance when compared to a commercially available 32‐channel head coil. Integration of the field camera was achieved without compromising the performance of either system. In vivo imaging with concurrent field monitoring enabled accurate spatiotemporal field corrections, significantly reducing geometric distortions, blurring, and ghosting in high b‐value DWI.

**Conclusion:**

The integration of high‐density MRI arrays with field monitoring systems facilitated the capture and correction of spatiotemporal field perturbations during strong gradient activity, substantially enhancing image quality and diffusion parameter mapping quality. These advancements provide a robust platform for exploring the structural intricacies of the human connectome.

## 1 INTRODUCTION

Magnetic resonance imaging stands at the forefront of neuroimaging technologies, offering unparalleled insights into the structural and functional architecture of the living human brain. The endeavor to map the human connectome has particularly benefited from advances in diffusion‐weighted imaging (DWI),^1^, ^2^, ^3^, ^4^, ^5^ which uniquely traces neural connectivity pathways in vivo.^6^, ^7^, ^8^, ^9^, ^10^, ^11^ The impressive high‐performance gradient technology of Connectome MRI scanners with gradient strengths ≥200 mT/m have propelled DWI into a new era, enabling ultra‐high b‐value diffusion imaging (>30 000 s/mm^2^) with sub‐millimeter isotropic resolution and promising a more detailed visualization of in vivo human brain tissue microstructure.^12^, ^13^, ^14^ Despite these technical advancements, achieving high‐resolution DWI of the whole brain with high b‐values requires optimization of imaging encoding strategies to maintain sufficient signal‐to‐noise ratio (SNR) and ensure a tolerable acquisition time for the human subject. Traditionally, the gradient coil has borne the primary burden of encoding spatial information in MRI. However, relying solely on gradient coil encoding becomes impractical, as it necessitates extended scanning times to acquire large, high‐resolution image matrices for whole‐brain mapping. To overcome these limitations, it is critical to share the encoding burden between the gradient coil and dedicated large channel‐count receiver arrays.^15^, ^16^, ^17^ This distribution of encoding responsibilities allows for highly accelerated in‐plane^18^, ^19^ and multi‐slice^20^, ^21^, ^22^ parallel imaging acquisition techniques, including advanced volumetric thin‐slab acquisition methods such as gSlider‐SMS,^23^ with minimal noise amplification during image reconstruction within the sub‐millimeter regime.

To fully leverage the benefits of strong diffusion‐sensitizing gradients, it is essential to overcome the inherent challenges posed by non‐linear spatiotemporal disturbances in the magnetic field due to eddy currents and concomitant fields (i.e., Maxwell terms^24^) during image data readout.^25^, ^26^ These spatiotemporal field variations depend on the amplitude and direction of the diffusion‐encoding gradients.^27^, ^28^ While spatially linear eddy current field patterns can be effectively minimized with gradient pulse pre‐emphasis techniques,^29^, ^30^, ^31^, ^32^ non‐linear eddy current fields of higher spatial order combined with non‐linear concomitant fields can neither be compensated nor easily corrected.^33^, ^34^, ^35^ Thus, these parasitic field perturbations lead to k‐space shifts that ultimately manifest as geometric distortions and blurring in the image domain.

The challenge of simultaneously acquiring highly accelerated, high b‐value diffusion data with sufficient SNR while concomitantly monitoring field dynamics remains a significant barrier to progress in high‐resolution DWI with strong diffusion‐sensitizing gradients. Addressing these challenges requires the strategic combination of advanced gradient technology with high‐density MRI receiver arrays and field monitoring systems.^36^, ^37^, ^38^ This approach enhances sensitivity and acquisition speed through highly parallel detection and allows real‐time monitoring and correction of the spatiotemporal dynamics of the magnetic fields. It holds promise to unlock the full potential of high‐resolution DWI acquisition with strong diffusion‐sensitizing gradients.

In this study, we designed, constructed, and validated two dedicated multichannel high‐density head coil arrays, each equipped with an integrated 16‐channel field monitoring system, to outfit the first‐ and second‐generation Connectome MRI scanners. These designs are not only a testament to the evolution of MRI coil technology but also a critical step forward in the quest to accurately depict and understand the intricacies of the human brain connectome.

## 2 METHODS

### 2.1 Coil design

Both Connectome head coil arrays were constructed on anatomically conforming helmets that provided the necessary structural support for the coil assembly. The loop elements were positioned based on hexagonal and pentagonal tiling patterns,^39^ with adjacent elements overlapping to minimize inductive coupling. To optimize the construction process, fixed positioning aids were integrated onto the surface of the coil formers, enabling the assembly of the coil arrays by simply snapping the copper wires into place. Preventing short circuits between the coil conductors at the crossovers required arranging loop elements in three staggered layers. Moreover, mounts for preamplifier boards and field probes were incorporated into the design to facilitate the precise placement of these components.

**FIGURE 1:**
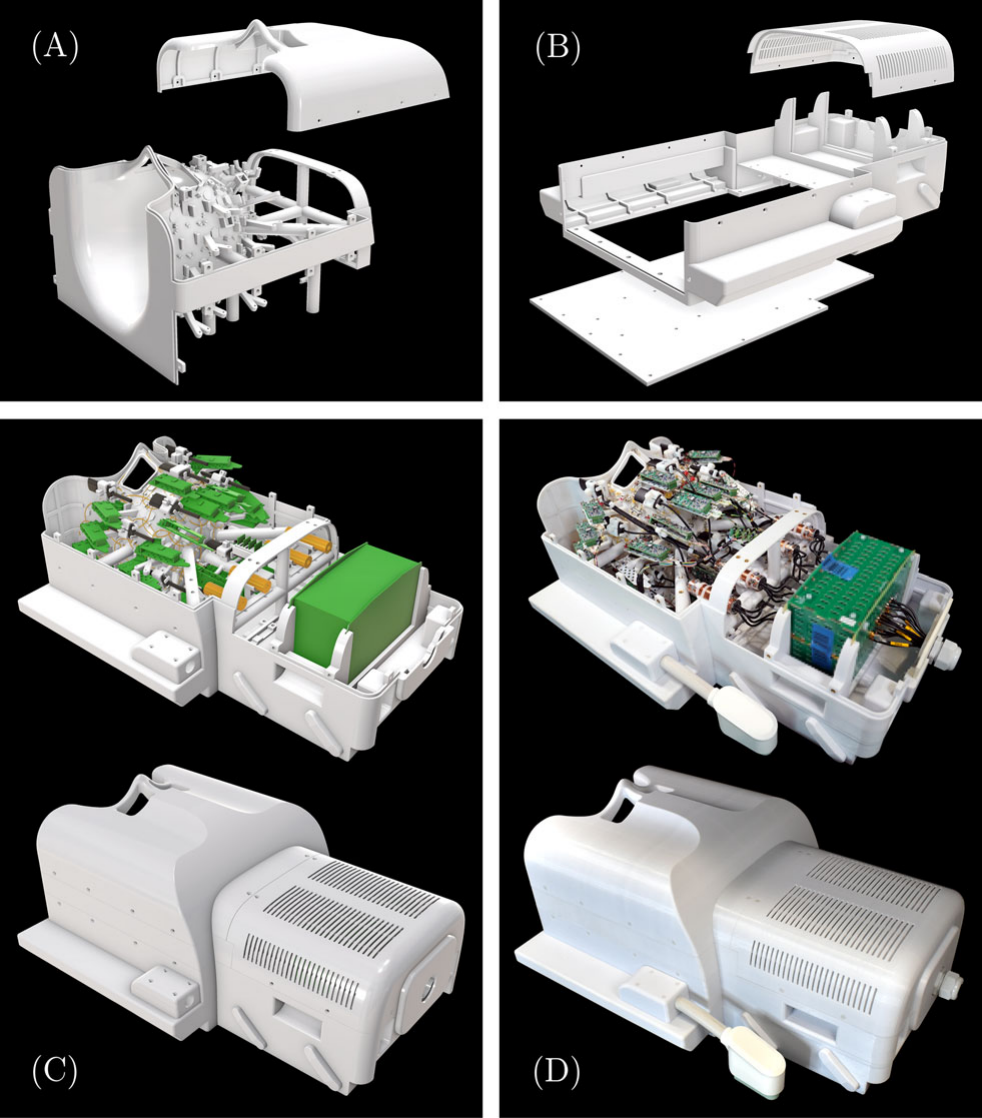
Design and construction of the 64‐channel Connectome 1.0 head coil (C1.64). (A, B) Mechanical coil components as a 3D CAD model. (C) CAD model including rendered electronic components for the Rx array and the field camera system. (D) Photo of the fully constructed 64‐channel head coil system with integrated 16‐channel field camera.

The receive (Rx) coil former of the Connectome 1.0 array consists of 64 loop elements with an average diameter of 63 mm. The coil former was incorporated into a base structure specifically tailored to fit the shape of the patient bed of the Connectome 1.0 scanner, enabling direct connection to the integrated RF sockets at the patient table's head‐end (Figure 1). The coil base additionally housed the front‐end electronics of the field camera and was optimized for the extensive cable routing of the receiver system. The anterior coil segment featured eye cutouts, ensuring both comfort and visibility during imaging sessions. A removable mirror can be installed to facilitate visual stimulus, enabling combined diffusion/fMRI protocols. The back‐end of the superior housing cover was outfitted with ventilation slots to ensure adequate cooling of the RF electronics during prolonged scans. The coil dimensions were 650 mm × 360 mm × 280 mm, with a total weight of 14.5 kg. We refer to this 64‐channel coil for the Connectome 1.0 scanner as C1.64.

**FIGURE 2:**
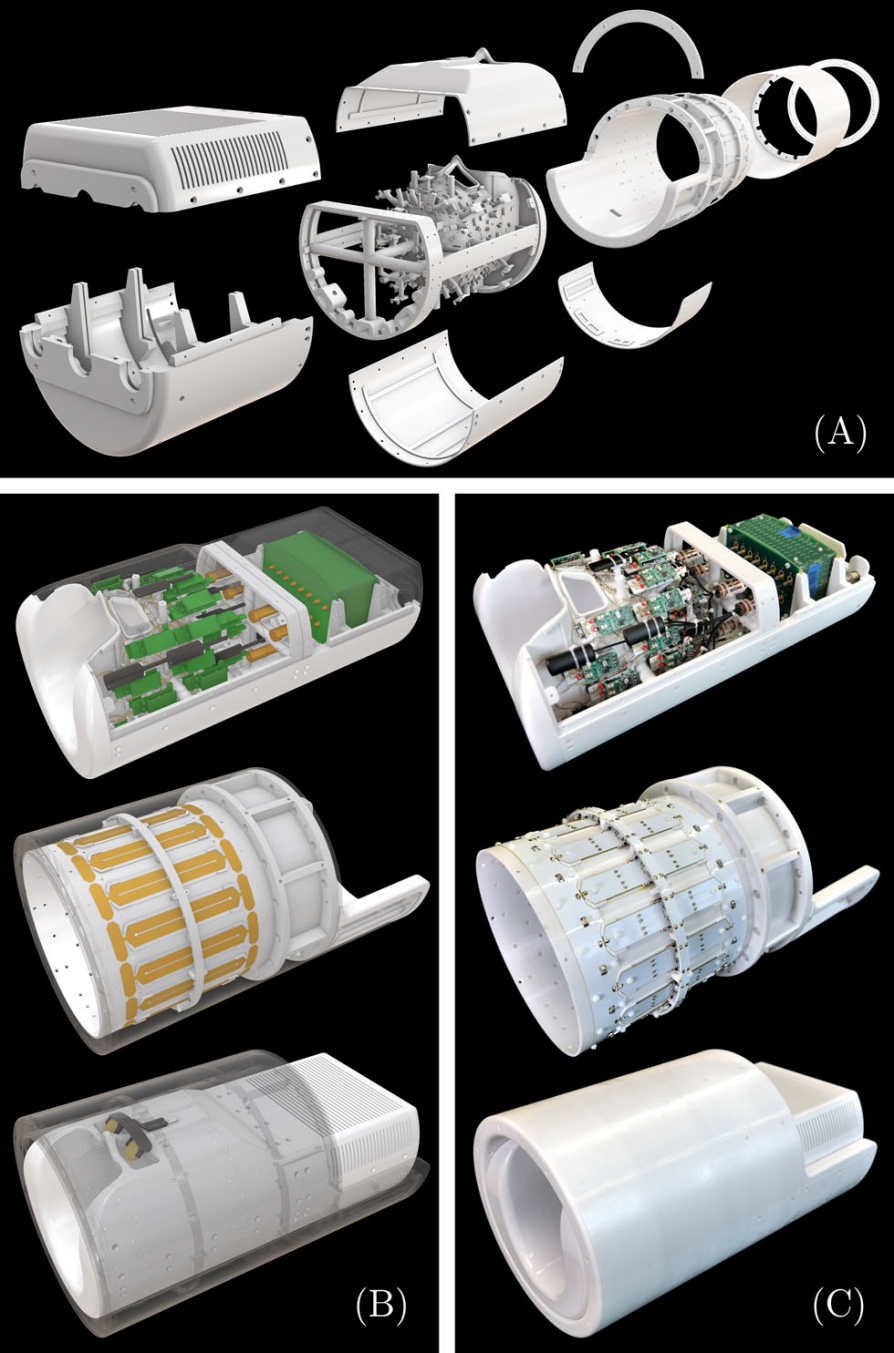
Design and construction of the 72‐channel Connectome 2.0 head coil (C2.72). (A) Mechanical coil components as a 3D CAD model. (B) CAD model including rendered electronic components for Rx array, field camera system, and Tx birdcage coil. (C) Photo of the fully constructed 72‐channel head coil system with integrated 16‐channel field camera and Tx birdcage coil.

The Connectome 2.0 scanner features a head gradient coil with a free bore diameter of 440 mm, which limits the radial space available for the head‐end of the patient bed, the transmit coil, and the receive coil system. The head‐end of the patient table accommodates the coil system and defines the maximum housing diameter of the Connectome 2.0 head coil assembly at 370 mm. The 72 Rx loop elements were distributed uniformly and overlapped with an average diameter of 58 mm. The Connectome 2.0 coil setup (Figures 2 and S1) required a local transmit (Tx) system, consisting of a birdcage coil structure and an RF shield (coil length = 235 mm, coil diameter = 317 mm, shield diameter = 363 mm). The tubular housing of the Tx coil was L‐shaped to allow an expanded visual field of view to the rear, facilitated by a mirror mounted between the Rx and Tx systems. The Connectome 2.0 RF coil system measures a radial diameter of 370 mm and a length of 650 mm, with a weight of 18.6 kg. We refer to this 72‐channel coil for the Connectome 2.0 scanner as C2.72.

All mechanical housing parts for both Connectome coils were designed using 3D CAD software (Rhino3D, Robert McNeel & Associates, Seattle, WA, USA) and 3D‐printed in polycarbonate plastics (Fortus 450mc, Stratasys, Ltd., Eden Prairie, MN, USA). Key components and features of the C1.64 and C2.72 coils are labeled in Figure S2. Photographs of the full scanner‐coil configurations are shown in Figure S3.

### 2.2 Receive coil arrays

The Rx circuitry chain closely followed our previous work (Figure S4).^16^, ^17^, ^40^, ^41^ Succinctly, we built each loop element for both Rx systems from 1.5mm‐thick silver‐plated copper wire. Minimizing resonance shifts and homogenizing the electric field along the loop coils required dividing the coil element into two segments. On one side, a tuning capacitor $C_{T3}$ was soldered, while on the other side, a subconnector facilitated the connection to the preamplifier daughterboard. The daughterboard included adjustable capacitors $C_{T4}$ and $C_{M}$ (GFX2700NM Sprague Goodman, Westbury, NY, USA) to fine‐tune each coil's resonance frequency and adjust its output impedance. A capacitive voltage divider $C_{T1}-C_{T2}$ (Series 11, Knowles Capacitors, Norwich, UK) provided RF balancing and was used to implement an active detuning circuit employing a variable inductance L (150‐02J08L, CoilCraft Inc., Cary, IL, USA) and a PIN diode D (MA4P4002B‐402, Macom, Lowell, MA, USA). By forward biasing the PIN diode, a parallel resonant circuit $C_{T2}-L$ inserted a high impedance in series with the coil loop, effectively suppressing current flow at the Larmor frequency during excitation. For enhanced subject safety, a redundant passive detuning circuit with a pair of cross‐parallel passive diodes $D_{X}$ (MADP‐011048‐TR3000, Macom, Lowell, MA, USA) was integrated. The combination of $C_{T1}$ and $C_{M}$ transformed the output of the coil elements to the noise‐matched impedance of 50 Ω. Simultaneously, they formed a parallel LC resonant circuit with the given inductive input impedance of the preamplifier to enable preamplifier decoupling.^42^ Each preamplifier daughterboard was equipped with a twin preamplifier (Siemens Healthineers, Forchheim, Germany) and connected to two adjacent coil elements. The preamplifier outputs were routed through cable traps to suppress common‐mode currents and prevent potential interactions with the transmission systems before being connected to the coil plugs.

Both Rx array coils were optimized through various RF bench measurements using a vector network analyzer (ZNB‐4, Rohde & Schwarz, Munich, Germany) with the coils being loaded with an anthropomorphic head‐neck‐torso phantom (26.8 L H_2_O, 33.5 g NiSO_4_·6 H_2_0, 134 g NaCl). A custom‐made switchable coil plug simulator was used to bias the PIN diodes and to provide the 3 V operating voltage required by the preamplifiers. For the RF bench tests, preamplifier dummy boards were employed for quickly assessing each receive element in terms of tuning, matching, and preamplifier decoupling.^40^ A 3‐way rotary switch on the dummy board allowed for quickly terminating the surface coils to either to (A) a 50 Ω impedance, (B) the complex input impedance of the preamplifier, or (C) an $S_{11}$ pass‐through connection to the vector network analyzer. The loop coils were tuned to 123.25 MHz and matched to 50 Ω through an $S_{11}$ measurement. Active detuning and preamplifier decoupling were verified by an $S_{21}$ measurement conducted with a double‐probe coupled to the coil element under test. Geometrical decoupling was assessed by directly measuring the $S_{21}$ parameters between two adjacent loops using the pass‐through connection of the preamplifier dummy board.

### 2.3 Transmit birdcage coil

**FIGURE 3:**
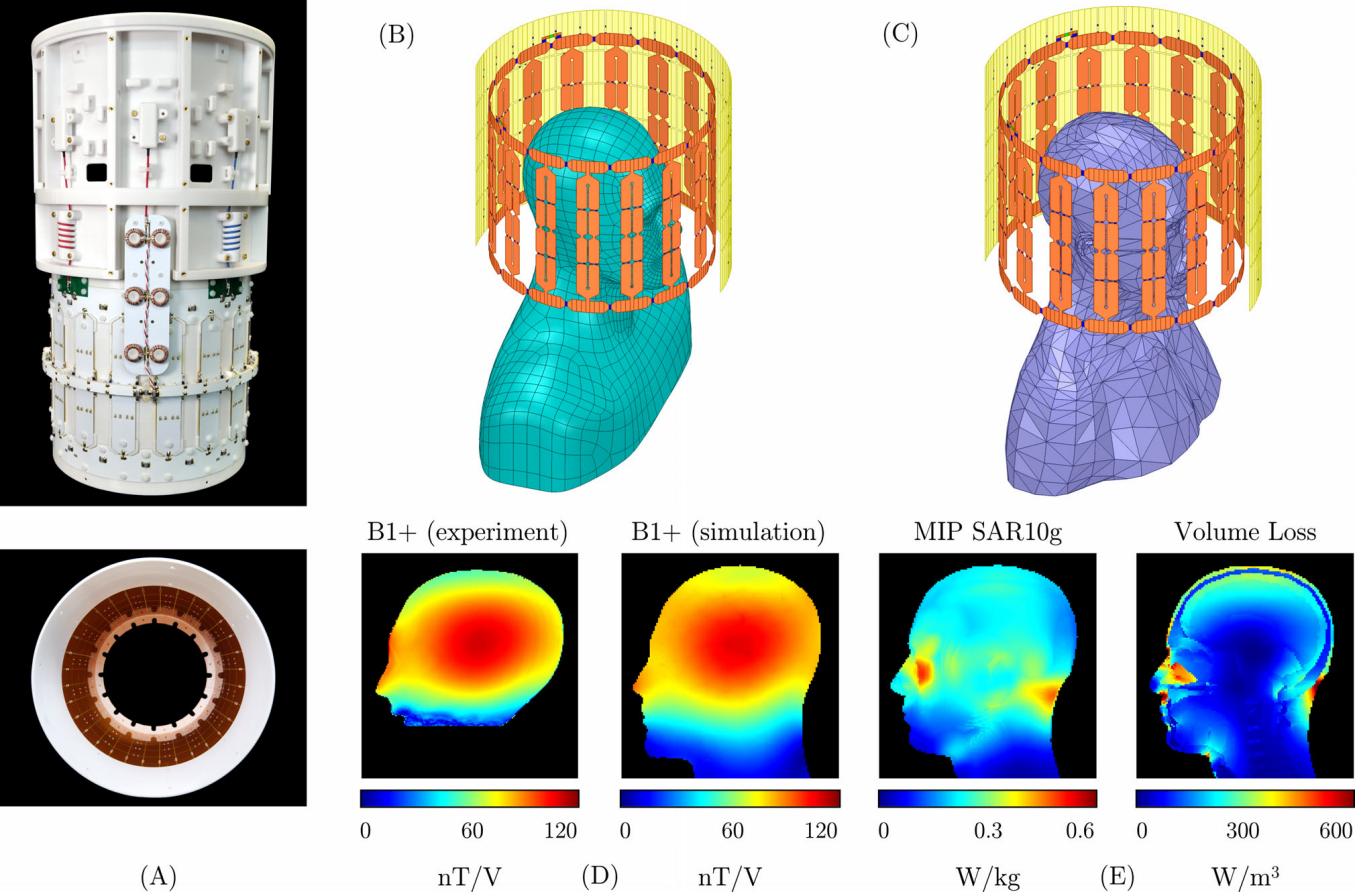
Design and simulation of the circularly polarized birdcage coil used in the Connectome 2.0 system. (A) The fully constructed birdcage coil and RF shield. (B) Simulation geometry of the birdcage coil loaded with an anthropomorphic head‐neck‐torso phantom. (C) Simulation geometry of the birdcage coil loaded with a multi‐tissue head‐neck‐torso model. (D) Comparison of the simulated and measured $B_{1}^{+}$ efficiency in the homogeneous phantom, with peak values of 109 nT/V (simulated) and 111 nT/V (measured). (E) Maximum intensity projection (MIP) of the 10g‐averaged SAR and the volume loss density, displayed in the central sagittal slice, both computed for the multi‐tissue head‐neck‐torso model. The maximum local SAR was 0.56 W/kg, and integration of the volume loss density yielded a total absorbed power of 0.75 W.

Whereas the Connectome 1.0 MRI scanner has its own built‐in transmit coil installed by the manufacturer, the new Connectome 2.0 MRI scanner requires a local Tx coil. This Tx coil was constructed as a circularly polarized bandpass birdcage coil (Figure 3). The copper structure was fabricated from FR4 printed circuit boards and comprised 16 rungs, two end‐rings, and a radially centered bias supply ring, all securely mounted on a tubular coil former and reinforced with epoxy resin. High‐power PIN diodes (MA4P7470F‐1072T, Macom, Lowell, MA, USA) were installed at the center of each rung and connected via surface mount RF chokes (1812CS‐272, CoilCraft Inc., Cary, IL, USA) to the bias supply ring. Each diode was forward‐biased by 155 mA for active tuning and inverse‐biased by −30 V for detuning. The bias ring was powered by a 5 A current source through a filter panel equipped with toroidal RF chokes to isolate the DC supply line from RF fields.

The coil was tuned using 43 pF capacitors (Series 25, Knowles Capacitors, Norwich, UK) in each end‐ring segment and 150 pF capacitors (Series 11, Knowles Capacitors) in a series/parallel combination in each rung (Figure S5). The transmit ports were matched to 50 Ω using a balanced capacitive network, while loaded with the same anthropomorphic head‐neck‐torso phantom. Quadrature port isolation was monitored using $S_{21}$, while $S_{11}$ measurements ensured proper tuning and matching. The coaxial cables of each drive port were passed through cable traps to block common‐mode currents. Suppressing eddy currents in the high‐performance gradient system required constructing the RF shield using segmented 9 μm copper foil with 1 nF capacitors soldered across the segments to provide RF continuity for mirror currents.

Numerical simulations conducted with HFSS (ANSYS Electronic Desktop 2021, ANSYS Inc., Canonsburg, PA, USA) supported the construction and validation of the birdcage coil. Two separate simulations were performed. The first simulation, utilized the digital replica of the actual anthropomorphic head‐neck‐torso phantom load (σ = 0.72 S/m, $\epsilon_{r}$ = 63.8), aimed to validate the birdcage coil model by comparing the simulated $B_{1}^{+}$ field distribution against actual measured data. This comparison confirmed the accuracy of the electromagnetic model, reinforcing confidence in its use for safety‐critical assessments. The second simulation employed a multi‐tissue head‐neck‐torso model to assess the safety parameter k (maximum 10g SAR per unit input power). This analysis calculated the 10g‐averaged SAR in accordance with IEEE C95.3‐2002 (R2008) standards.^43^ A conservative estimation of k was obtained by calculating the absorbed RF power through numerical integration of the simulated volume loss density in MATLAB (MathWorks, Natick, MA, USA), instead of using the total input power.

### 2.4 Field camera

A commercial field camera (Skope Magnetic Resonance Technologies AG, Zurich, Switzerland) equipped with 16 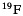 NMR field probes was integrated into each Connectome coil array. Optimizing the positioning of the field probes was critical for two key reasons.

**FIGURE 4:**
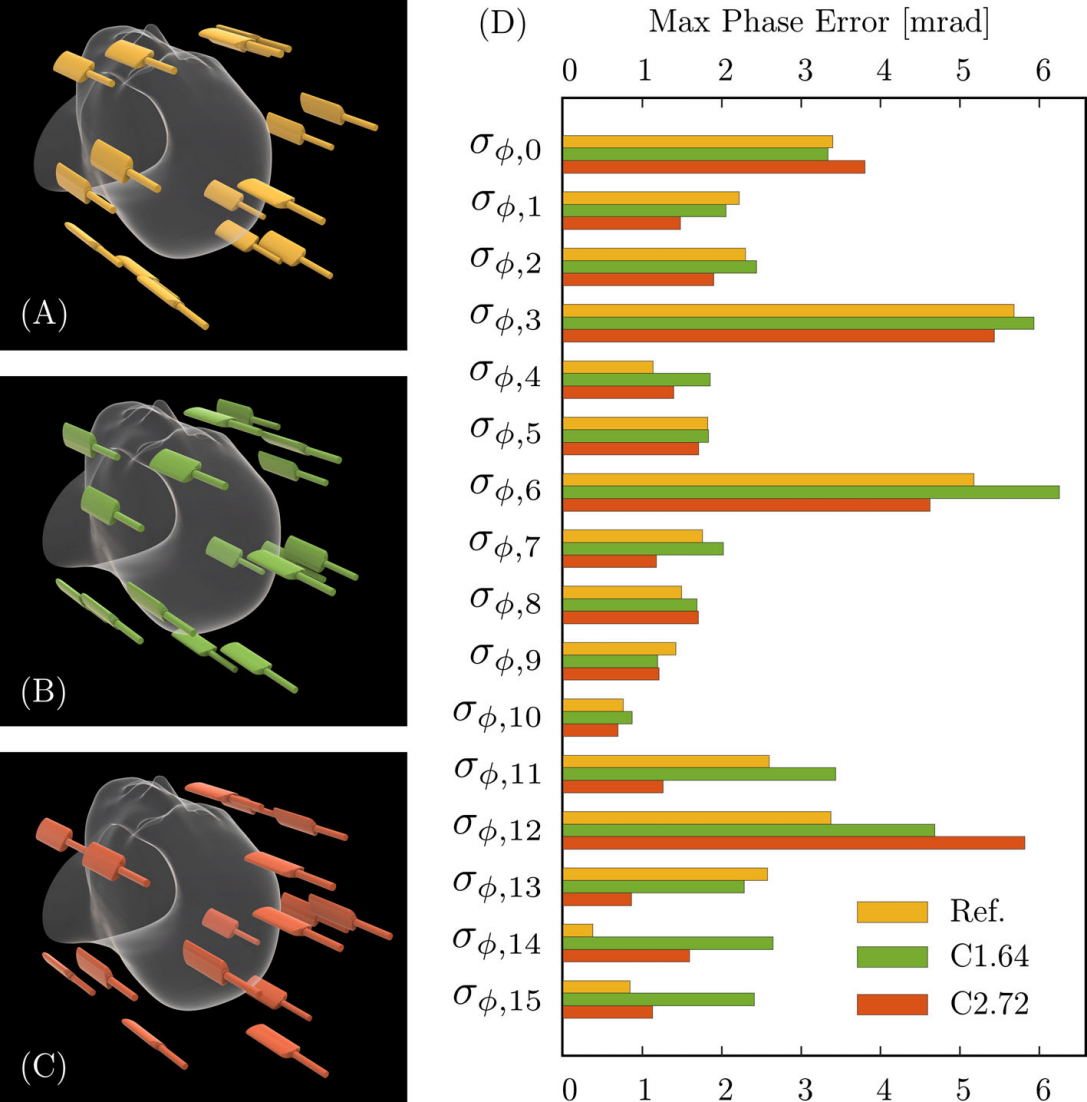
Field probe positioning. (A) Standard field probe configuration for head imaging (three z‐stacked rings with 4, 6, and 5 probes, and one at the tip). (B) Field probe distribution in the C1.64 coil (three z‐stacked rings with 4, 6, and 5 probes, and one at the tip). (C) Field probe distribution in the C2.72 coil (three z‐stacked rings with 4, 7, and 4 probes, and one at the tip). (D) Calculated maximum phase errors for the phase coefficients up to the third order. The total phase errors were 44.9 mrad for the C1.64 coil, 35.8 mrad for the C2.72 coil, and 36.9 mrad for the reference distribution.

First, the dynamic phase coefficients derived from the probe signals are sensitive to noise propagation, which can affect their reliability.^44^ The spatial arrangement of the probes directly influences the spherical harmonics expansion used for coefficient calculation, necessitating precise placement. While increasing probe separation can theoretically reduce noise propagation,^33^ space constraints in high‐density multichannel arrays limit this approach. Additionally, positioning probes too far from the isocenter increases the risk of signal loss due to spin dephasing under strong gradient pulses. To address these challenges, we employed an iterative repositioning process (Figure 4), starting with a standard four z‐stacked ring configuration (4‐6‐5‐1 probes).^45^ Each distribution was evaluated for maximum phase error $\sigma_{\phi ,\lambda ,\max}$ within a 10 cm radius sphere using the formalism from Barmet et al.^45^ For the Connectome 1.0 coil, this process resulted in an optimized 4‐6‐5‐1 distribution with an average probe distance of 12.9 cm from the isocenter. Similarly, the Connectome 2.0 coil achieved an optimal 4‐7‐4‐1 distribution, with probes positioned at an average distance of 14.1 cm from the isocenter.

Second, mitigating signal degradation caused by the exponential decay of the FID signal was essential. This decay, dictated by the $T_{2}$ relaxation time of the fluorine droplets, is further accelerated by spatially variable susceptibility.^44^ Once the SNR falls below a critical threshold, phase information from the FID can no longer be reliably extracted, leading to complete signal loss. To address this, state‐of‐the‐art field probes^46^ employ susceptibility‐matched materials to minimize local field inhomogeneities. Additionally, we ensured that each NMR‐active droplet within the probes was positioned approximately 2 cm from surrounding materials, further reducing the risk of signal degradation. Although the relatively large probe housing initially presented challenges for placement within the dense coil arrays, it provided a benefit by allowing secure fixation of the probes without affecting the fluorine droplets themselves. This careful positioning prevented contact with the sharp edges of the polycarbonate coil formers, where we previously observed significant signal losses in our experimental setups (Figure S6).

The field probe cables were equipped with ^1^H floating RF traps^47^ to suppress common‐mode currents and connected to a shielded electronics module, containing T/R switches, low‐noise preamplifiers, and excitation power amplifiers. This module box was also accommodated within each coil array housing. The routing of the field camera output cables varied between the two MRI systems: With the Connectome 1.0 coil, the cable bundle was loosely routed through the magnet bore towards the filter panel of the MRI scanner's shielded room. For the Connectome 2.0 coil, the camera's cable bundle was plugged directly into the head‐end of the patient table and routed through the integrated MRI system's cabling chain to the filter panel. The field camera's NMR spectrometer console was situated in the equipment room outside the MRI cabin.

During initial performance evaluations of the C1.64 coil with integrated field probes, a $B_{1}^{+}$ focusing effect exceeding 10% was observed. To address this, the receive coil wiring was revised, and additional cable traps were strategically installed to suppress common‐mode currents and mitigate the focusing effect. Flexible bazooka baluns^48^ were added to the cables of the field probes, and circular six‐layer baluns^49^ were installed on the Rx cables of the coil arrays. These adjustments effectively prevented the focusing effect, restoring uniform $B_{1}^{+}$ field distribution while maintaining the system's overall performance.

**FIGURE 5:**
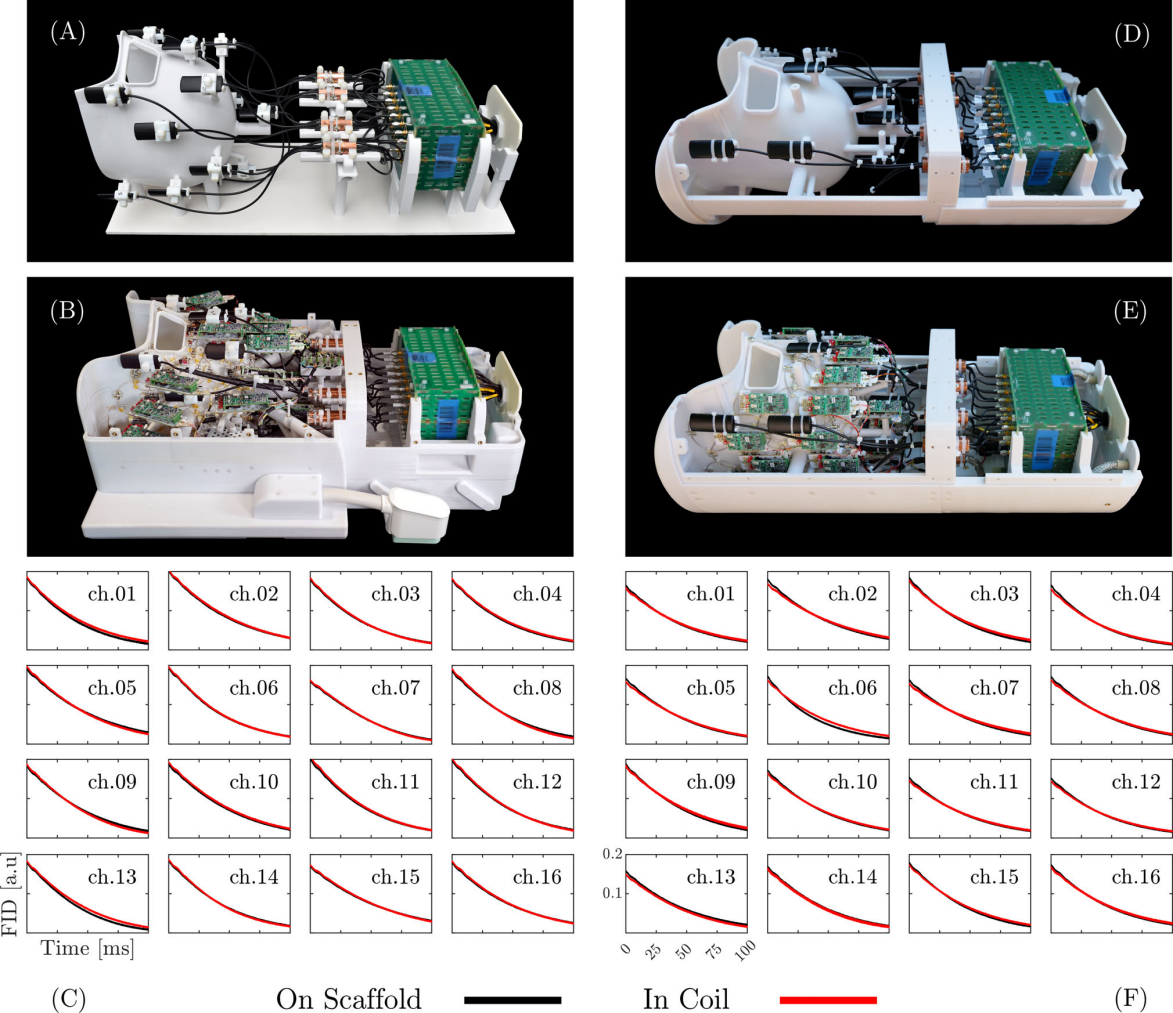
Field probe FID measurements. (A, D) Field probes with shielded front‐end electronics installed on blank coil formers (scaffold). (B, E) Field probes and RF electronics installed in the C1.64 and C2.72 coils, respectively. (C, F) FID line plots of the individual field probes when incorporated on coil arrays (red) and on the scaffolds (black). The FID lifetimes of the field probes remained unaffected after installation into the coils.

The lifetime characteristics of each field probe's FID signal were analyzed and compared under two conditions: With the field camera directly integrated within the array coil structures, and with the field camera mounted on an isolated scaffold holder without any array coil electronics present (Figure 5). The design of the scaffold probe holder ensured that the field probes, cable traps, and module box were positioned identically in both sets. During the field probe's FID measurements, an anthropomorphic head phantom was used as a load, and $B_{0}$ shimming was performed.

### 2.5 Coil evaluation

Imaging was carried out using the 3 Tesla Connectome 1.0 scanner (MAGNETOM Skyra Connectom A, Siemens Healthineers, Forchheim, Germany) and the 3 Tesla Connectome 2.0 scanner (MAGNETOM Connectom.X, Siemens Healthineers, Forchheim, Germany), equipped with the 64‐channel coil and the 72‐channel coil, respectively. The Connectome 1.0 system features a whole‐body gradient coil capable of reaching a maximum gradient strength of 300 mT/m and a maximum slew rate of 200 T/m/s.^50^ In contrast, the Connectome 2.0 system is equipped with a head‐only gradient coil capable of achieving a maximum gradient strength of 500 mT/m and a maximum slew rate of 600 T/m/s.^51^ The Connectome 2.0 gradient system is powered by two gradient power amplifiers per axis (peak current = 1 200 A, peak voltage = 2 250 V), as opposed to four power amplifiers per axis in the Connectome 1.0 system (peak current = 900 A, peak voltage = 2 250 V).

Before and after integration of the field probes, phantom measurements were conducted to assess the potential impact of the field cameras on the imaging performance of the constructed array coils. We carried out comparative analyses of imaging metrics, focusing on SNR as a measure of available receive sensitivity, $B_{1}^{+}$, to evaluate potential field focusing or damping that occur in the presence of the field camera as well as examining inter‐receive element noise correlations. These metrics were compared with a commercially available 32‐channel Rx head coil (Siemens Healthineers, Forchheim, Germany). These data were obtained from gradient echo images (TR/TE = 200 ms/4.8 ms, FA = 15°, matrix size = 192 × 192, FoV = 256 mm × 256 mm, slice thickness = 6 mm, bandwidth = 200 Hz/pixel, avg. = 6) with and without (noise only data) RF excitation. The SNR maps were calculated using a covariance‐weighted root sum‐of‐squares reconstruction following the Kellman method.^52^ To account for different transmit coils, the SNR maps were normalized against the actual flip angle maps. The flip angle maps were obtained using the double‐angle method^53^ from gradient echo images. Subsequently, $B_{1}^{+}$ maps were derived from the flip angle maps based on the magnitude integral of the RF pulse and the transmission voltage.^54^ SENSE g‐factor maps^18^ were computed to evaluate the encoding performance of the array coils. To assess intersubject variability in coil performance, we conducted additional in vivo scans with the C1.64 and C2.72 coils, each using a group of four subjects and identical imaging protocols as described above. These measurements enabled evaluation of SNR reproducibility across varying head sizes and positions within the coils.

### 2.6 Diffusion imaging and field monitoring

Diffusion imaging and field monitoring experiments were conducted on the Connectome 1.0 and Connectome 2.0 scanners, utilizing their respective custom‐developed head coils. In vivo human imaging was conducted on four subjects in compliance with protocols approved by the local institutional review boards (IRB), and written informed consent was obtained from all participants before data acquisition.

For both systems, diffusion imaging employed fast spin‐echo sequences modified to incorporate arbitrary readout gradients and field monitoring triggers. The Connectome 1.0 system utilized a twice‐radially undersampled spiral trajectory, with fully sampled k‐space achieved by acquiring each slice with a second spiral arm rotated by 180°. The same diffusion protocol was also applied to a phantom filled with low‐diffusion liquid^55^ to evaluate field monitoring performance in a controlled setting. In contrast, the Connectome 2.0 system used EPI with Cartesian k‐space sampling, with diffusion‐weighted imaging performed at b‐values progressively increasing to 30 000 s/mm^2^. To allow direct comparison across different gradient strengths, additional acquisitions were performed using the Connectome 2.0 scanner configured to emulate the Connectome 1.0 gradient performance. Accelerated multi‐slice diffusion imaging was further explored on the Connectome 2.0 system using a multiband (MB) factor of 2 and a GRAPPA in‐plane acceleration factor of 2; a reference acquisition without multiband excitation was also performed. High‐resolution tractography was performed on the Connectome 2.0 system using a multi‐shell acquisition protocol. Additionally, gradient‐echo images were acquired on both systems to generate $B_{0}$ and coil sensitivity maps, with sequence parameters adjusted to align with the FoV and resolution of the diffusion acquisition. During all acquisitions, field probes dynamically captured trajectories up to the third order. A summary of the acquisition parameters is provided in Tables S1 to S4.

Raw data and trajectory information from both scanners were processed in real time using vendor‐provided software. Scanner corrections, such as $B_{0}$‐eddy current and zero‐order Maxwell compensation, were removed to avoid double correction. This involved protocol simulation to determine the nominal phase correction, which was then applied using custom scripts to remove phase contributions from each k‐space line. Non‐linear gradient corrections were not applied to the voxel‐wise b‐value calculations. Diffusion tensor metrics, including mean diffusivity (MD), fractional anisotropy (FA), and principal eigenvectors, were computed using MRtrix3.^56^ White matter (WM) regions were extracted with SPM12,^57^ and fiber orientation distributions (fODF) were reconstructed with multi‐shell multi‐tissue constrained spherical deconvolution (MSMT‐CSD).^58^, ^59^ Probabilistic tractography was performed, with results visualized using MRtrix3 and DSI Studio,^60^ enabling detailed white matter exploration and connectivity analysis. Additional details on image reconstruction using dynamically monitored trajectories can be found in Ramos‐Llordén et al.^28^

## 3 RESULTS

A detailed summary of the essential RF bench‐level metrics for the two constructed coils is presented in Table S5. The $Q_{\text{UL}}$/$Q_{L}$ ratio for the C1.64 array (63 mm elements) was 3.5, while the C2.72 array (58 mm elements) showed a Q‐ratio of 3.4. Measurements were made on the populated array, with all elements actively detuned except the one under test. Phantom loading caused resonance frequency shifts between 0.2 to 0.3 MHz, confirming sufficient distributed tuning capacitors to minimize electric losses. All elements achieved matching levels below −18 dB at 123.25 MHz. Bench tests obtained from both coils showed a range of decoupling between nearest‐neighbor elements, from −12 to −22 dB, with an average of −16 dB. The coupling between next‐nearest neighbors (nonadjacent pairs) ranged from −10 to −27 dB, with an average of −17 dB for both coils. Preamplifier decoupling contributed an additional −18 dB isolation. Active PIN diode detuning provided isolation of −37 dB. The implemented cable traps in the Rx systems provided a mean common‐mode rejection of −22 dB, while the field camera system suppressed common modes by an average of −18 dB.

Figure 3 illustrates the electromagnetic simulation results for the bandpass birdcage coil in the Connectome 2.0 system. The simulated $B_{1}^{+}$ field for a homogeneous phantom showed a peak value of 109 nT/V at the center, validating the measured value of 111 nT/V (Figure 3D). This close agreement in both peak values and spatial field distribution of $B_{1}^{+}$ confirms the simulation's accuracy. This validation provides confidence in safety‐critical simulations using realistic human models, which are essential for safety assessment. Figure 3E shows simulations with the realistic human model, revealing a maximum 10g‐averaged local SAR of 0.56 W/kg and total absorbed power of 0.75 W. The safety parameter k was calculated as 0.75 kg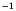. An additional safety factor of 2 was included to provide a conservative estimate of local SAR.

The iterative field probe placement process aimed to minimize phase errors while balancing practical constraints imposed by coil geometry. Figure 4 illustrates the final optimized probe distributions for the C1.64 and C2.72 coils, compared with the reference configuration. The C1.64 array showed maximum phase errors from $\sigma_{\phi ,10}=0.9$ mrad to $\sigma_{\phi ,6}=6.3$ mrad, while the C2.72 had maximum phase errors from $\sigma_{\phi ,10}=0.7$ mrad to $\sigma_{\phi ,12}=5.8$ mrad. Both implemented field camera systems closely matched the reference distribution values ($\sigma_{\phi ,14}=0.4$ mrad to $\sigma_{\phi ,3}=5.7$ mrad). Total accumulated phase errors were 44.9 mrad, 35.8 mrad, and 36.9 mrad for C1.64, C2.72, and reference configurations, respectively.

To further assess the impact of the field probe integration, Figure 5 shows FID signal measurements, which remained nearly identical across both Connectome coils, regardless of the presence of the receiver coil arrays. The FID signals exhibited an average lifetime (1/e of maximum values) of approximately 50 ms, persisting for at least 100 ms before reaching the noise floor. This consistency confirms minimal influence of susceptibility‐induced local field inhomogeneities on probe performance.

**FIGURE 6:**
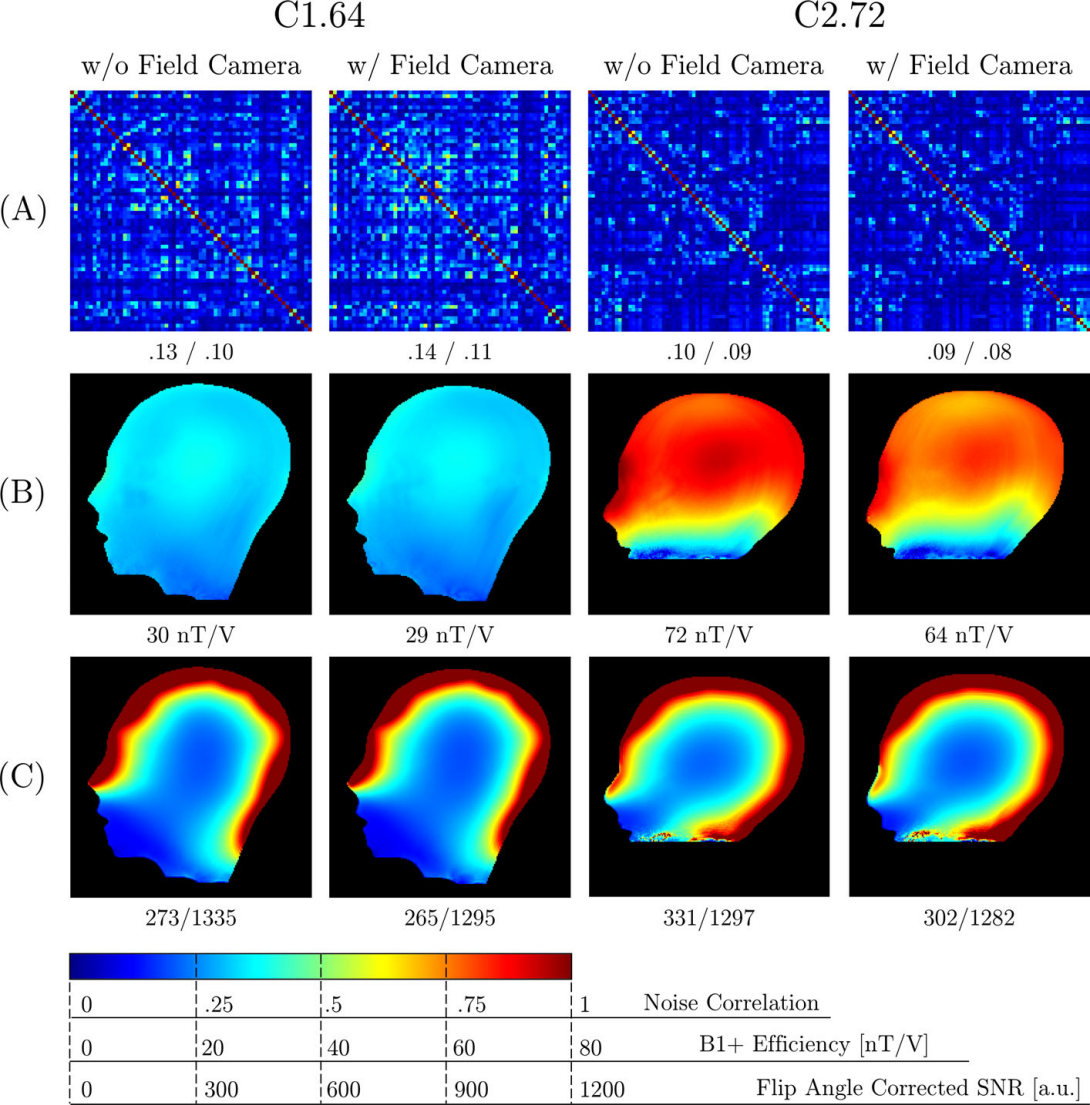
Coil‐Camera interaction. (A) Noise correlation matrices, (B) $B_{1}^{+}$ efficiency maps, and (C) Flip angle‐corrected SNR maps for both the C1.64 and C2.72 coil systems. All measurements were performed with and without the field camera system integrated. The values below each plot represent the mean and standard deviation of noise correlation, the mean $B_{1}^{+}$ efficiency at the phantom center, and the mean SNR in the central and peripheral phantom regions.

The effect of field probe integration on coil performance is summarized in Figure 6, which compares noise correlation, $B_{1}^{+}$ efficiency, and flip angle‐corrected SNR maps before and after probe integration. The C1.64 coil showed a minor increase in mean noise correlation from 13% to 14% post‐integration, while the C2.72 coil showed a decrease from 10% to 9%. The $B_{1}^{+}$ field at the phantom's center decreased from 30 nT/V to 29 nT/V in C1.64 and from 72 nT/V to 64 nT/V in C2.72. Field probe integration resulted in modest SNR reductions: C1.64 showed uniform 2.9% decreases in both center (273 to 265) and periphery (1 335 to 1 295), while C2.72 experienced an 8.8% reduction in center SNR (331 to 302) and a minimal 1.2% decrease in peripheral SNR (1 297 to 1 282).

**FIGURE 7:**
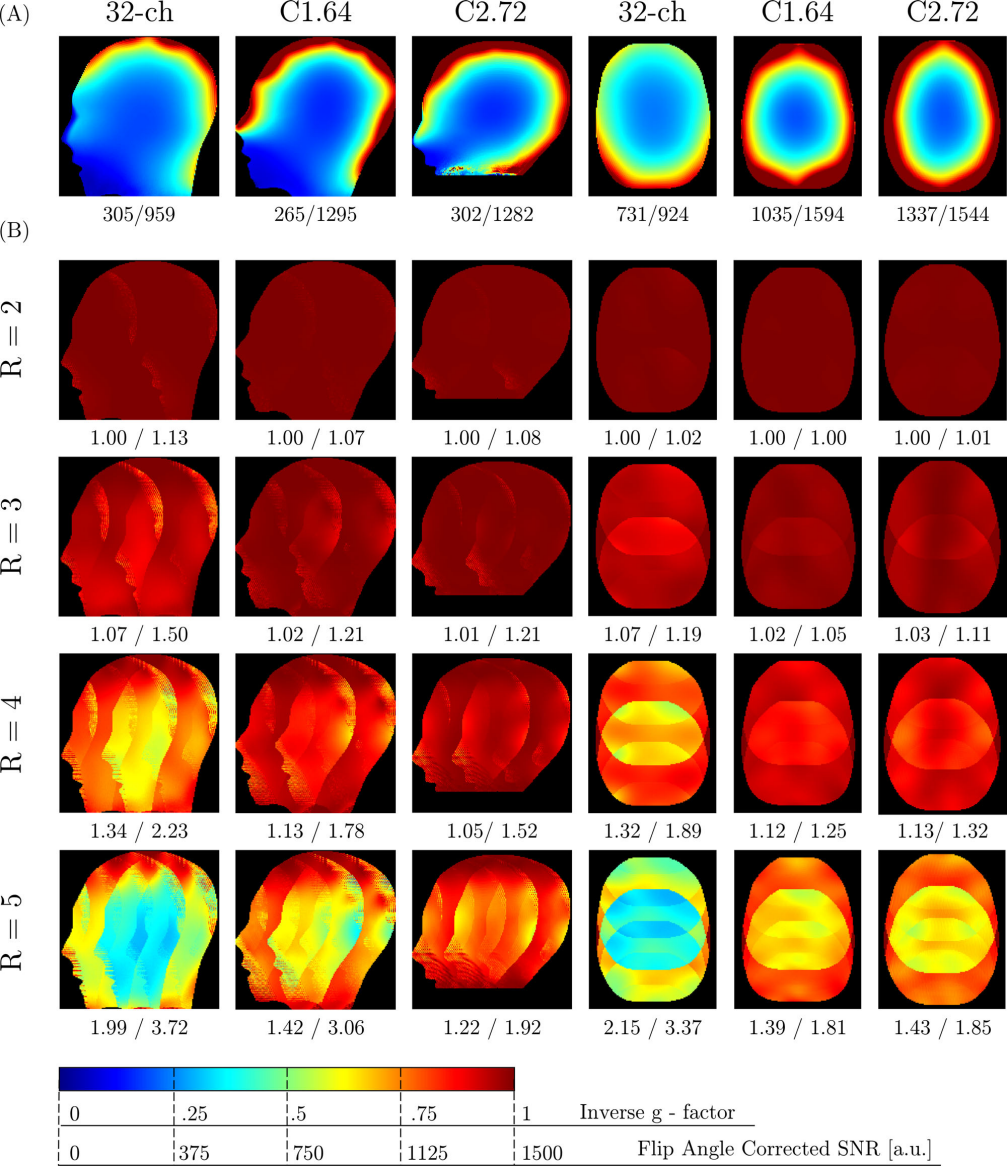
Coil performance comparison. (A) Flip angle‐corrected SNR maps for the commercial 32‐channel, C1.64, and C2.72 head coils, shown for both sagittal and transverse slices. The values below each plot represent the mean SNR in the central and peripheral regions of the phantom for the sagittal slice, and in the left‐right and anterior‐posterior regions for the transverse slice. (B) Inverse g‐factor maps for the corresponding coils and slices, computed for acceleration factors R = 2, 3, 4, and 5. The values below each plot indicate the mean and maximum g‐factor across the entire slices.

Figure 7 presents comparative analyses of SNR and inverse g‐factor maps of the 32‐channel commercial coil and both Connectome coils. In sagittal slices, the Connectome coils demonstrated superior performance in the periphery, with mean SNR values of 1 295 (C1.64) and 1 282 (C2.72) compared to 959 for the 32‐channel coil. However, in the center, the 32‐channel coil led with an SNR of 305, followed by C2.72 (302) and C1.64 (265). Transverse SNR maps highlight the advantages of the high‐density Connectome coils. In the left‐right regions, C2.72 achieved the highest SNR (1 337), outperforming both the 32‐channel coil (731) and C1.64 (1 035). In the anterior‐posterior regions, C1.64 and C2.72 also outperformed the 32‐channel coil (924), with SNR values of 1 594 and 1 544, respectively.

The g‐factor analysis across relevant acceleration factors demonstrated lower mean and maximum g‐factors for the Connectome coils compared to the 32‐channel coil, indicating enhanced parallel imaging performance. In sagittal slices at R = 5, C2.72 achieved the lowest g‐factors (mean: 1.22, max: 1.92), outperforming both C1.64 (mean: 1.42, max: 3.06) and the 32‐channel coil (mean: 1.99, max: 3.72). This observed performance advantage also remained at lower R‐values. In transverse slices at R = 5, C1.64 performed best, with a mean and maximum g‐factor of 1.39 and 1.81, respectively, followed by C2.72 (mean: 1.43, max: 1.85), both surpassing the 32‐channel coil (mean: 2.15, max: 3.37).

Figures S7 and S8 present the in vivo SNR maps and corresponding line profiles obtained from four subjects using the C1.64 and C2.72 coils, respectively. For both coils, the SNR profiles show comparable values in central brain regions across all subjects. Inter‐subject variability becomes more apparent in peripheral areas, particularly in subjects with smaller head circumferences, where the increased distance from the coil elements results in lower SNR. Subject 4 in the C1.64 dataset shows a marked reduction in SNR in the upper brain region, likely due to suboptimal positioning. Overall, the spatial SNR distribution patterns are consistent across subjects, supporting the robustness of the coil designs for reliable signal reception across varying head sizes and placements.

In summary, the Connectome 1.0 and 2.0 coils demonstrated enhanced performance over the 32‐channel coil in both SNR and g‐factor metrics. SNR measurements revealed approximately 1.4‐fold higher values averaged across all regions and slices. The improved g‐factor performances at higher acceleration factors enabled the Connectome coils to achieve an additional level of acceleration, while maintaining noise levels comparable to the 32‐channel coil.

**FIGURE 8:**
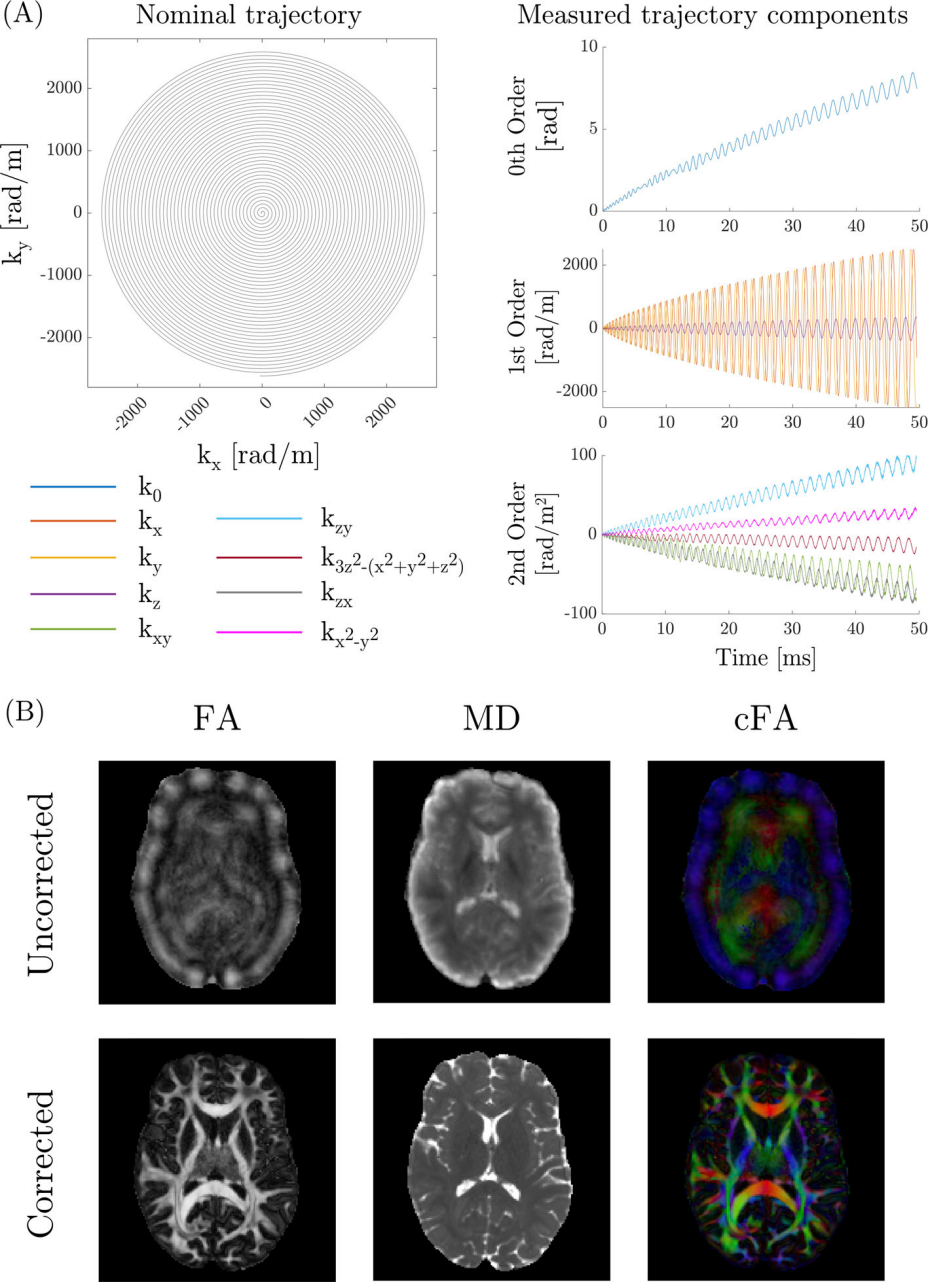
Concurrent field monitoring on Connectome 1.0. (A) Nominal and measured spiral trajectories (for the first arm of the first slice) for the 1.2mm spiral acquisition using the C1.64 coil on the 3T Connectom 1.0 scanner. The field probes in the coil allowed measurement of trajectories up to the second order. The measured trajectory shows some $k_{z}$ components due to the tilted field of view. (B) Comparison of diffusion‐derived measures FA ([0, 1] windowing), MD ([0.1, 2.0] μm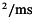 windowing), and cFA when the trajectory of the first slice is used for all slices (top row), and when the full dynamic trajectories are used (bottom row). The dynamic correction gave rise to crisp parameter maps, whereas the uncorrected maps were blurry.

Figure 8 demonstrates the advantage of concurrent field monitoring during diffusion‐weighted spiral acquisitions with the Connectome 1.0 system. Figure 8A shows both the nominal and the measured k‐space trajectories for the first arm of the spiral acquisition. The measured trajectories exhibit small $k_{z}$ components due to the FoV being tilted in the transverse plane. The observed image blurring is attributed to a combination of concomitant gradient fields during readout and long‐lived eddy currents induced by the diffusion‐weighting gradients that introduce higher‐order field deviations. These results emphasize the precision of the integrated field probes in detecting subtle trajectory deviations during dynamic scanning. Figure 8B compares the MD, FA, and cFA maps under two conditions: Applying the trajectory of the first slice for all slices (top row) versus utilizing full dynamic trajectories (bottom row). Without dynamic correction, the parameter maps appear blurred. In contrast, applying dynamic trajectory corrections significantly enhanced the sharpness and clarity of the blurred appearance of uncorrected maps, validating their importance for diffusion tensor imaging fidelity. The same effect was observed with the phantom filled with the low‐diffusion liquid (Figure S9).

**FIGURE 9:**
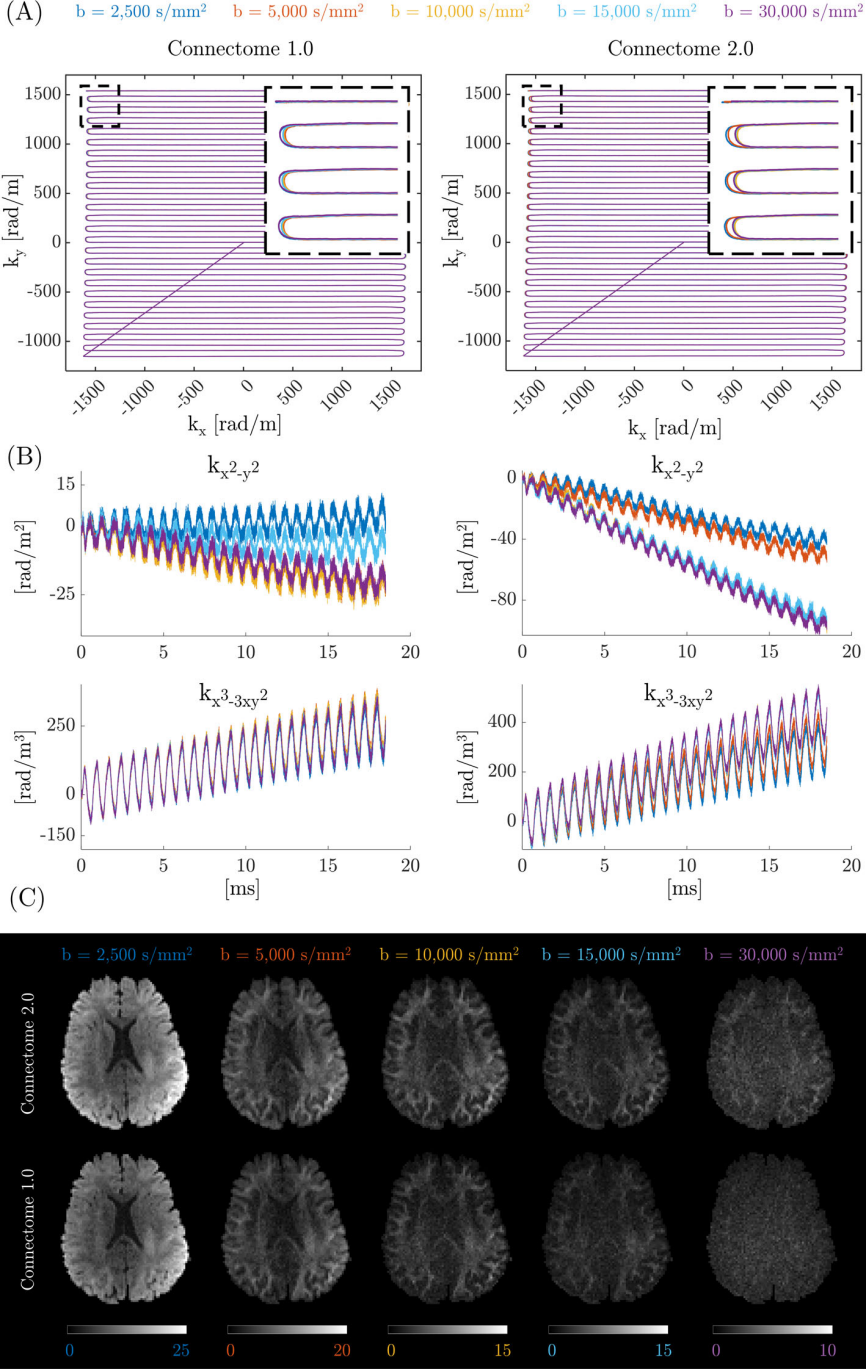
Concurrent field monitoring on Connectome 2.0. (A) Measured k‐space trajectories for representative diffusion‐weighted images acquired at different b‐values. The trajectories exhibit variations between b‐values, attributed to eddy currents induced by diffusion gradients. (B) Higher‐order phase terms (up to third order) also show significant variation across b‐values, underscoring the importance of concurrent field monitoring. (C) By incorporating spatiotemporal phase variations measured through the concurrent field monitoring system into the image reconstruction framework, images can be reconstructed without visible artifacts. The trajectories and images labeled “Connectome 1.0” were recorded on the Connectome 2.0 scanner using gradient settings emulating the Connectome 1.0 system.

Figure 9 highlights the necessity of concurrent field monitoring for high b‐value diffusion MRI using the Connectome 2.0 scanner. Long‐decay eddy currents induced by diffusion gradients persist during image encoding. With the same diffusion gradient direction but varying b‐values, these eddy currents significantly alter the k‐space trajectory. Additionally, higher‐order phase terms, which are particularly pronounced with the Connectome 2.0 scanner, vary depending on the b‐value. This underscores the importance of concurrent field monitoring to correct diffusion‐dependent higher‐order phase errors for each diffusion‐weighted image. By incorporating these higher‐order phase terms into the image formation model and solving the reconstruction as an inverse problem, it is possible to achieve high‐fidelity, artifact‐free, ultra‐high b‐value diffusion MRI. To complement the representative results shown in Figure 9, Figure S10 presents axial slices acquired across all six non‐collinear diffusion directions at b=2500 s/mm^2^. The full‐brain coverage demonstrates consistent image quality and confirms the robustness of the field monitoring corrections across different diffusion encodings. In addition, Figure S11 illustrates accelerated multi‐slice diffusion imaging with the C2.0 system, comparing axial slices acquired with and without simultaneous multi‐slice (SMS) acquisition. Comparable anatomical coverage and image quality were achieved, with the SMS acquisition reducing scan time by half.

**FIGURE 10:**
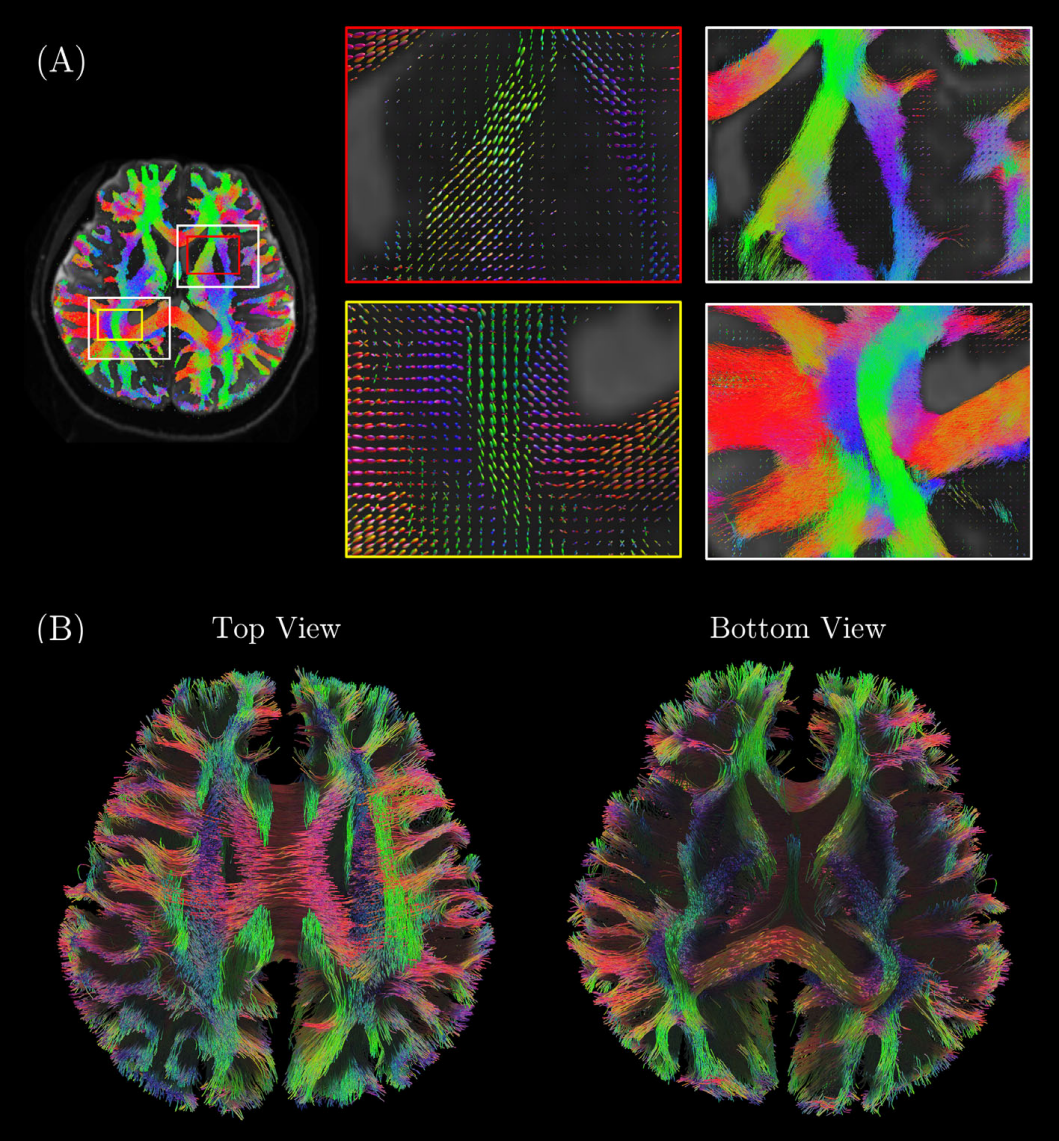
High‐resolution diffusion tractography and fiber orientations on Connectome 2.0. (A) WM tracts overlaid on fiber orientation distribution. The red box highlights the region where the anterior limb of the internal capsule and the external capsule are located. The yellow box highlights the region where the superior longitudinal fasciculus, posterior thalamic radiation, and body of corpus callosum are located. (B) WM tracts in axial top and bottom views, mapped using DSI‐Studio.

Figure 10 shows WM fiber tracts superimposed on fiber orientation distribution in (A) and 3D rendered WM fiber tracts in (B) obtained from high‐resolution diffusion MRI data reconstructed based on concurrent field monitoring. Clear discrimination of fiber tracts, where fiber crossing occurs, can be observed in both the regions where the anterior limb of the internal capsule and the external capsule meet (red box in Figure 10A) and where the posterior thalamic radiation and superior longitudinal fasciculus (SLF) meet (yellow box in Figure 10A). The 3D‐rendered fibers in both the top and bottom views show fewer artifacts from ghosting or eddy current distortions (Figure 10B). SLF, corona radiata, and the body of the corpus callosum can be seen in the top view. Internal/external capsules and genu/splenium of the corpus callosum can be seen in the bottom view.

## 4 DISCUSSION

In this study, we designed, constructed, and validated two high‐density multichannel MRI detector arrays with integrated field monitoring systems for the Connectome 1.0 and 2.0 scanners, demonstrating substantial advancements in diffusion‐weighted imaging performance. The developed systems achieved significant improvements in SNR and g‐factor metrics compared to a commercially available 32‐channel head coil, while maintaining robust dynamic field monitoring capabilities. The observed SNR patterns between the different coil configurations reflect inherent trade‐offs in high‐channel coil design, including sizing differences of the detector arrays' geometry. The slightly lower central SNR in the high‐density arrays, compared to the 32‐channel coil, is a common phenomenon in high channel‐count arrays. Theoretically, central SNR should be comparable (central ultimate SNR in a spherical coil array geometry is adequately achieved with approximately 12 elements^61^), but the increased number of loop elements in higher channel‐count arrays contributes to an overall higher resistive component in the detection system. However, for in vivo diffusion imaging, accelerated SNR performance is particularly relevant, with both developed coils demonstrating their strengths due to enhanced encoding capabilities (lower g‐factors). Acceleration is crucial for in vivo diffusion imaging; it enables higher spatial resolution within clinically feasible scan times. Furthermore, parallel imaging techniques allow acquisition of more directional measurements within the same total scan time, thus enhancing the angular resolution necessary for advanced fiber tracking techniques.

A critical challenge in integrating field monitoring capabilities into high‐density receiver arrays was balancing two competing requirements: Field coefficient determination demanded field probe positioning that minimizes noise propagation in the spherical harmonic expansion, while simultaneously requiring each probe to operate in an environment free from susceptibility‐induced field distortions. These requirements often conflicted in the densely packed C1.64 and C2.72 receiver arrays, where the optimal geometric configurations for minimizing noise coefficients frequently positioned probes near receiver array components, potentially compromising the longevity of the FID signal. Our iterative optimization process successfully balanced these competing demands. The final probe arrangement enabled real‐time tracking of spatiotemporal magnetic field perturbations while minimizing noise propagation in field coefficient determination up to the second and third order (Figures 8 and 9). Careful positioning avoided proximity to the sharp edges of the polycarbonate coil formers and other receiver array components, which had caused significant signal degradation during earlier tests. This optimization ensured FID signal lifetimes of up to 100 ms for each field camera probe, enabling reliable phase extraction throughout k‐space sampling, even during diffusion‐weighted acquisitions with strong gradient pulses inducing parasitic non‐linear field distortions.

The overall observed difference in $B_{1}^{+}$ efficiency between the Tx coils is attributed to the geometric coil configurations used with the two Connectome systems. The Connectome 1.0 scanner uses a regular body coil for Tx with larger radial space to the subject, resulting in an overall lower $B_{1}^{+}$ efficiency, whereas the Connectome 2.0 scanner employs our custom‐built substantially smaller local birdcage Tx coil with reduced radial space to the subject, which enables a stronger $B_{1}^{+}$ field using the same input power. While the integration of field cameras into the coil arrays generally had a moderate impact on imaging performance, noticeable effects on the $B_{1}^{+}$ field were observed. With the C2.72 coil, a dampening effect of approximately −11% was observed. In contrast, the C1.64 coil initially exhibited a $B_{1}^{+}$ focusing effect, which was addressed through targeted post‐integration modifications. Revisions to the receive coil wiring and the addition of cable traps effectively suppressed common‐mode currents, restoring uniform $B_{1}^{+}$ field distribution and ensuring consistent system performance. Field alterations appear to have been driven primarily by common‐mode currents along the RF cables, which generated their own fields influencing $B_{1}^{+}$ efficiency. Depending on the specific wiring configuration, these currents could result in either field focusing or attenuation. Similar effects have been observed in previous studies; Gilbert et al.^37^ reported $B_{1}^{+}$ efficiency changes ranging from −30% to +15% in individual channels of a parallel transmit coil, while Schmidt et al.^38^ described alterations of up to ±10% in commercial coils integrated with field probes. According to the relationship $SAR\propto |B_{1}^{+}{|}^{2}$, the observed reduction in $B_{1}^{+}$ efficiency with the C2.72 coil should not inherently pose a safety risk. However, the current literature lacks systematic investigations into how complex cable routing might generate localized SAR elevations through common‐mode currents. Therefore, we have taken a conservative approach by treating the $B_{1}^{+}$ attenuation as analogous to $B_{1}^{+}$ focusing and have accordingly increased the safety margin of the simulated k factor. Conversely, the $B_{1}^{+}$ focusing effect initially observed in the C1.64 coil could potentially generate localized SAR hotspots, resulting in increased RF heating in specific regions. This issue was completely resolved through the strategic placement of cable traps and modifications to the cable routing.

The integration of high‐performance gradients into MRI systems enables advanced diffusion imaging but inherently generates non‐linear spatiotemporal field perturbations. Addressing these challenges requires an optimized interplay of technologies: Powerful gradients enable high b‐value diffusion imaging with high resolution, while high‐density receiver arrays deliver essential SNR and encoding capabilities for accelerated acquisitions. The integrated field camera system continuously monitors gradient‐induced distortions, capturing the dynamic field variations from eddy currents and concomitant fields.

This hardware study demonstrated that the synergistic integration of high‐performance gradients with optimized RF technology for signal reception and field monitoring has proven essential for achieving high‐quality, high‐resolution diffusion imaging. High‐performance gradient MRI systems remain unable to realize their full potential when not synergistically paired with optimized high‐density RF arrays and concurrent field monitoring. The diffusion‐weighted imaging experiments demonstrated that combining highly parallel signal reception with continuous k‐space trajectory corrections throughout diffusion acquisition yielded sharp and accurate DWI parameter maps. This successful implementation establishes a robust platform for advanced diffusion imaging applications.

## 5 CONCLUSION

We successfully developed and validated two high‐density multichannel MRI detector arrays with integrated field monitoring capabilities for the Connectome 1.0 and 2.0 scanners. The combination of high‐density receiver arrays with concurrent field monitoring proved particularly valuable for diffusion‐weighted imaging applications. Dynamic trajectory measurements captured subtle k‐space sampling deviations during intense gradient activity, while real‐time field corrections substantially improved diffusion parameter map quality. This integrated approach effectively addresses the technical challenges of high‐resolution diffusion imaging with strong gradients, where spatiotemporal field perturbations can severely impact image quality. These technological developments represent a crucial step forward in human connectome mapping. By enabling high‐fidelity in vivo diffusion imaging, these developments provide a robust foundation for studying the intricate structural connectivity of the human brain.

## CONFLICTS OF INTEREST STATEMENT

The Max Planck Institute for Human Cognitive and Brain Sciences and Wellcome Centre for Human Neuroimaging have institutional research agreements with Siemens Healthcare. NW holds a patent on acquisition of MRI data during spoiler gradients (US 10,401,453 B2).

## Supporting information


**Data S1.** Supporting Information.

## Data Availability

Data supporting the findings of this study are openly available in GitHub at https://github.com/keyarray/connectinvivocoil.^62^
