# Supplementary material for: High‐density MRI coil arrays with integrated field monitoring systems for human connectome mapping
Source: Magn Reson Med. 2025 Jun 18;94(5):2286–303. doi: 10.1002/mrm.30606 (PMC12393205; doi:10.1002/mrm.30606)
Supplement: Supplementary file 1 — Data S1. Supporting Information. [file MRM-94-2286-s001.pdf]

Supporting Information Figures

High-Density MRI Coil Arrays with Integrated Field Monitoring Systems for Human Connectome Mapping

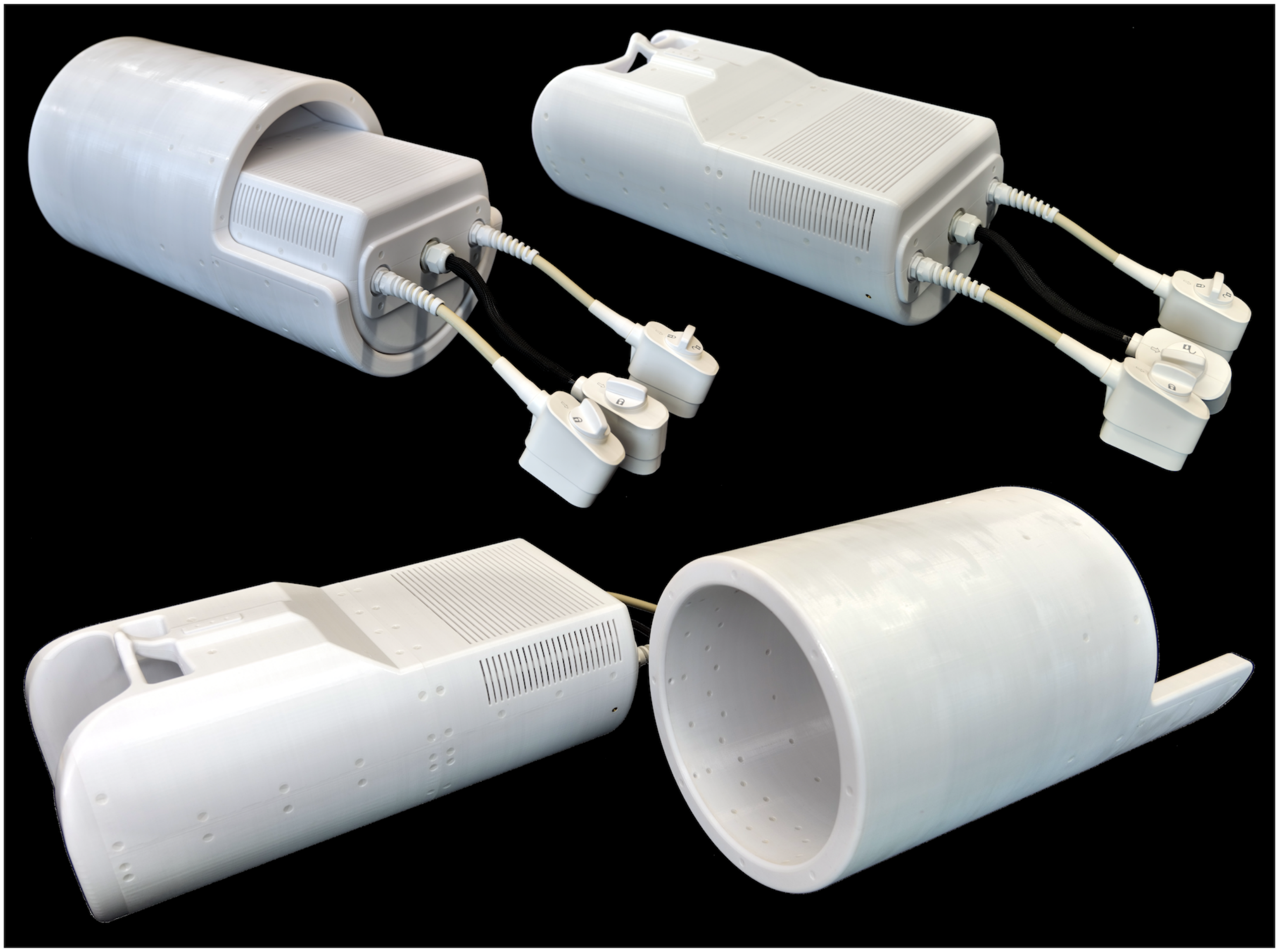

Figure S1: Fully assembled Connectome 2.0 coil (C2.72). The coil incorporates three RF plugs: one central plug dedicated to the field monitoring system, and two additional plugs for the Rx and Tx coil systems. Each of coil system plugs accommodates 36 Rx lines and one Rx line.

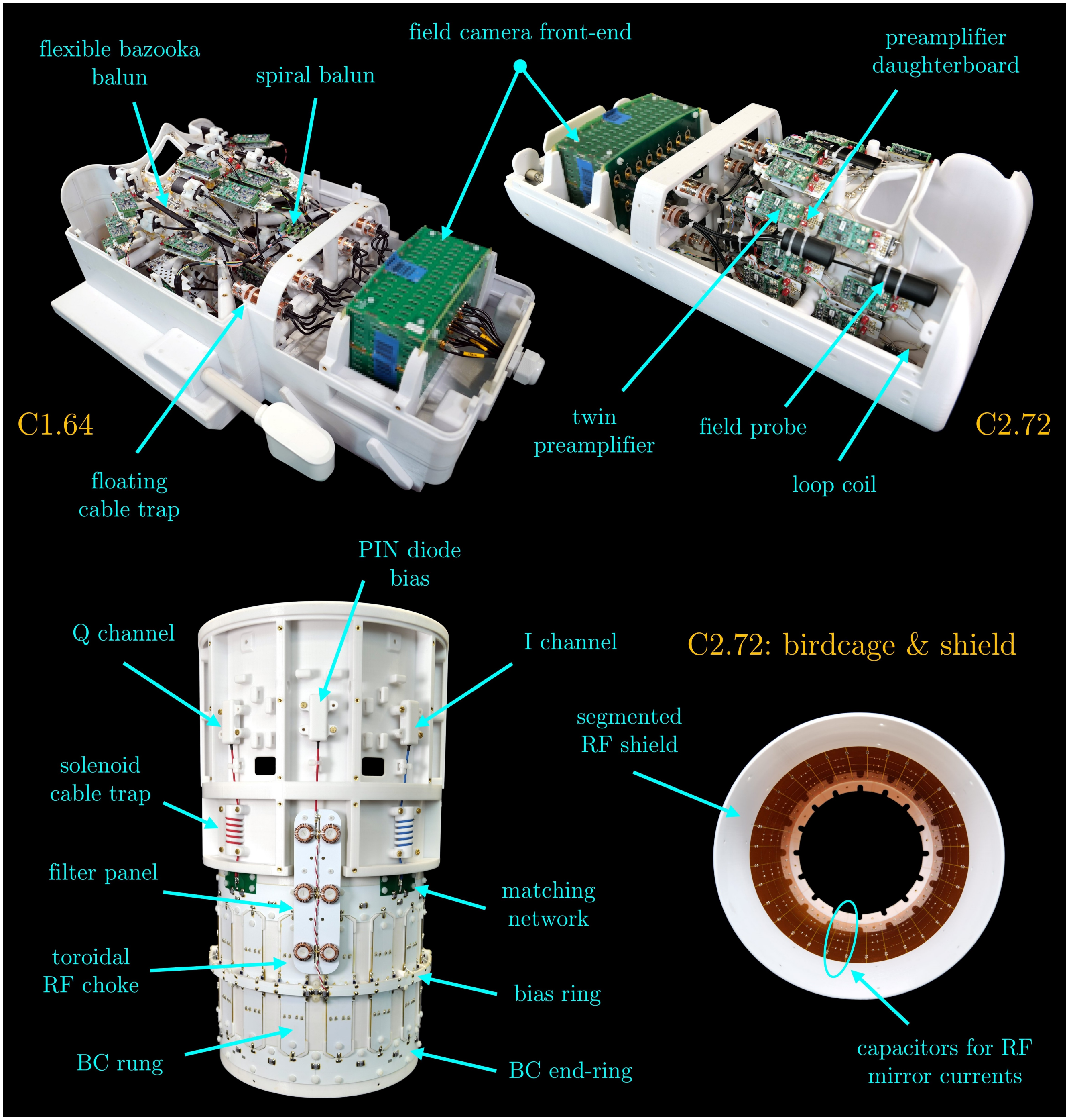

Figure S2: Detailed view of the C1.64 and C2.72 coil systems showing the labeled components of both receive arrays and the C2.72 transmit architecture. The annotated photographs highlight key elements including the receive loops, preamplifiers, field monitoring hardware, cable traps, and baluns. Also visible are various transmit components such as the segmented RF shield, matching network, PIN diode bias circuitry, and RF choke structures.

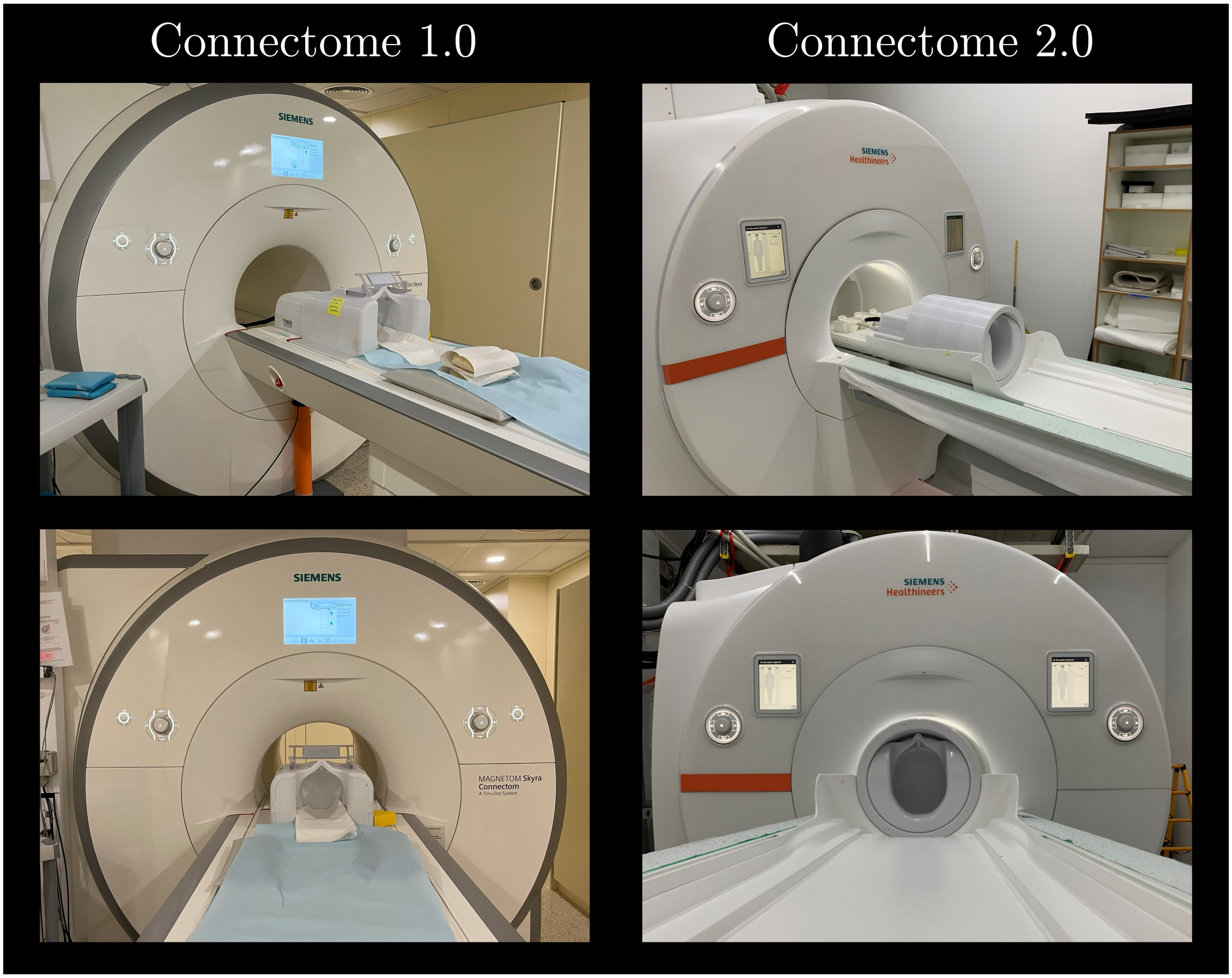

Figure S3: Comparison of the Connectome 1.0 and Connectome 2.0 scanners with their respective developed coil arrays C1.64 and C2.72 installed. The side-by-side views illustrate key differences between the systems, including variations in bore diameter, patient table design, and coil interfacing.

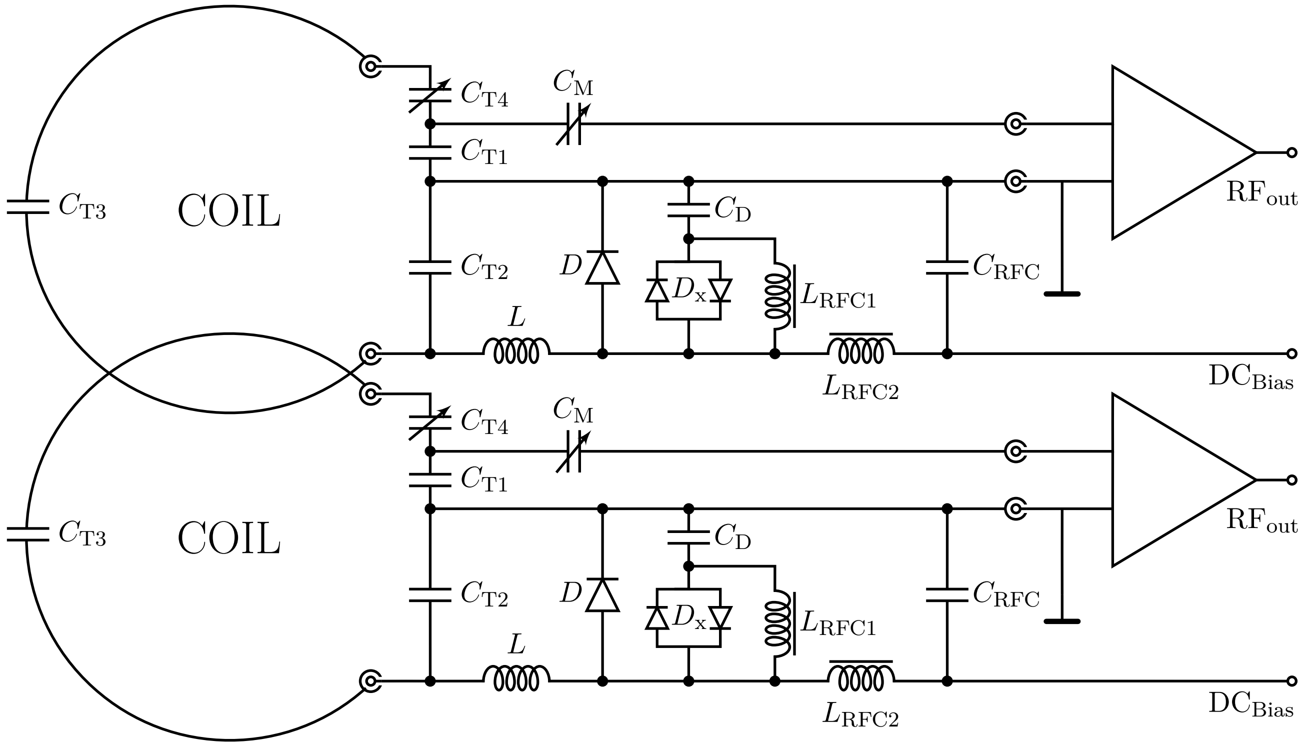

Figure S4: Circuit schematic for a representative Rx twin coil element and preamplifier chain. The loop coil element contains four tuning capacitors ($C_{T1} - C_{T4}$). A suitable combination of C_{T1} and C_M allows impedance matching to $50\,\Omega$ under loaded condition. An active detuning trap is formed around C_{T2} using the inductor L and a PIN Diode D. The matching network and the detuning trap are located on the separate daughterboard of the preamplifier, which connects the coil loop elements using a board-to-board connector. Component values for a 63 mm diameter coil element: : $C_{T1} = 47$ pF, $C_{T2} = 56$ pF, $C_{T3} = 27$ pF, $C_{T4} = 18$ pF, $C_M = 15$ pF, $C_D = 180$ pF, $C_{RFC} = 2.2$ nF, $L = 25$ nH, $L_{RFC1} = L_{RFC2} = 2.7$ µH.

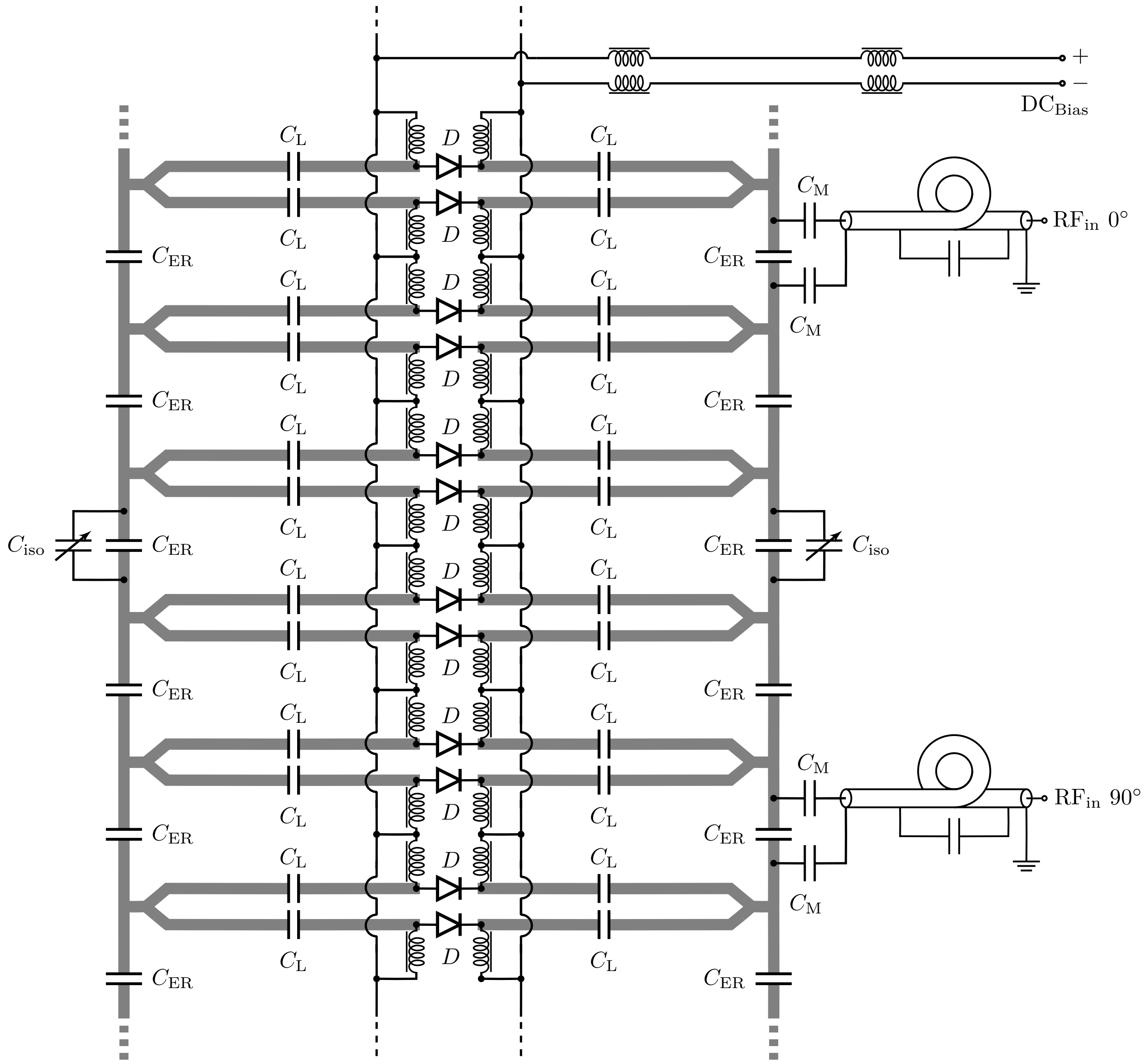

Figure S5: Circuit schematic for the circularly polarized Tx birdcage coil. The band-pass birdcage coil consists of 16 rungs (6 are shown). The coil has an active tuning mechanism, by forward biasing two PIN diodes (MA4P7470F-1072T, Macom, Lowell, MA) in each leg. The PIN diode system operates with a forward bias of 5 A (155 mA each diode) for active tuning and a reverse bias of –30 V for detuning. The tuning network consists of 43 pF end-ring capacitors (Series 25, Knowles Capacitors, Norwich, UK), while eight 150 pF capacitors (Series 11, Knowles Capacitors) are arranged in a series/parallel combination within each leg ($C_L = 150pF \,||\, 150pF = 300pF$). Two series capacitors C_M in each port match the loaded coil's impedance to 50 Ω. To improve S_{21} port isolation, C_{iso} was incorporated.

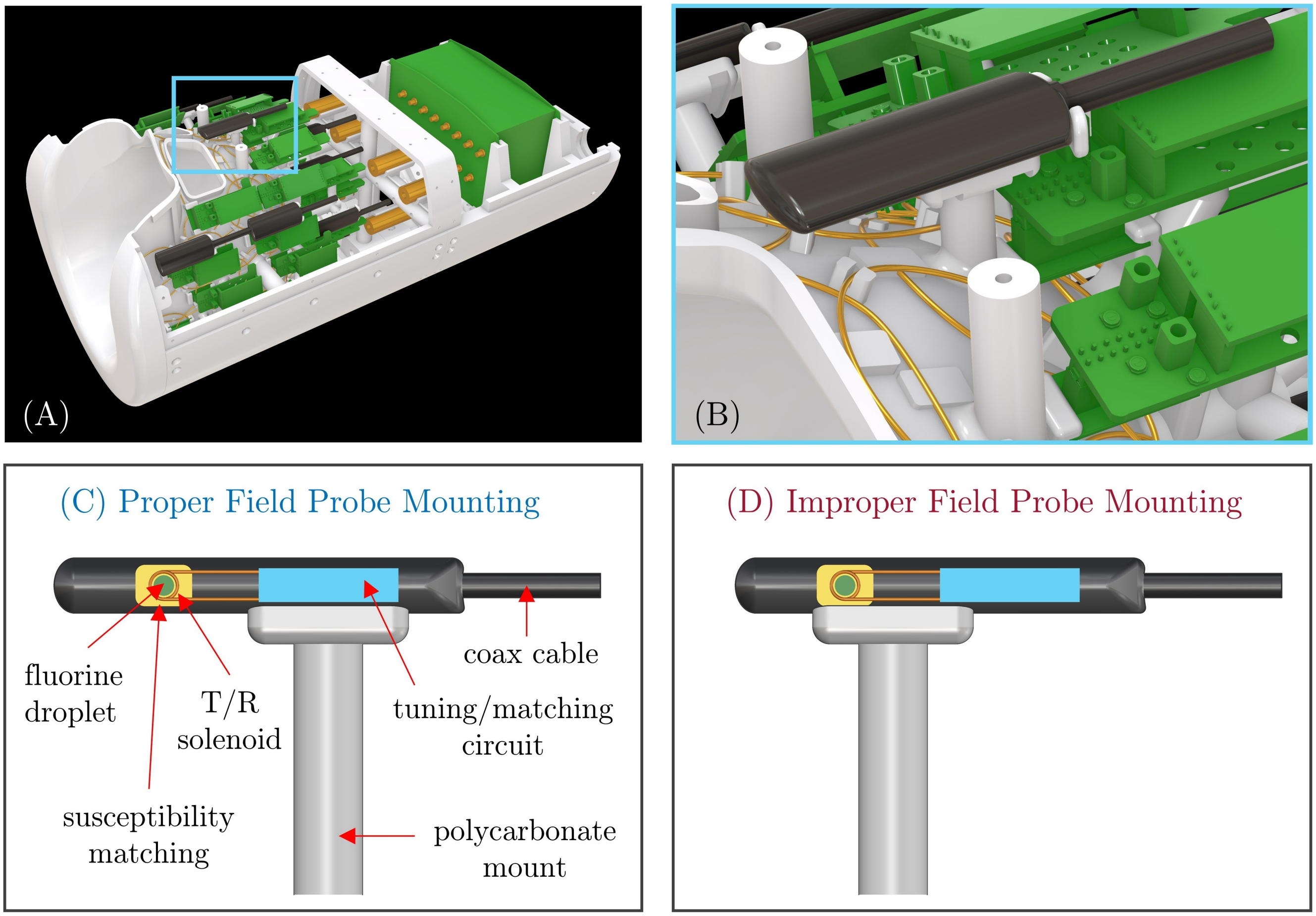

Figure S6: Spatial design constraints and field probe mounting. (A) CAD rendering of the C2.72 coil, including RF components and structural elements. The blue rectangle indicates the zoomed-in region shown in (B). (B) Close-up view of the coil interior showing the three staggered loop layers, a mounted field probe, preamplifier boards and their daughterboards, and the polycarbonate mounting structures. The Rx elements are arranged in three staggered layers to prevent short circuits at the crossovers; the height difference between the first and third layer is minimal (3.5 mm), with 1 mm clearance between layers and 1.5 mm copper wire thickness. This view illustrates the spatial constraints encountered during the probe integration process. (C) Schematic illustration of a properly mounted field probe, showing key components (e.g., fluorine droplet, T/R solenoid, matching circuit) and appropriate spacing from surrounding structures. (D) Schematic illustration of an improper mounting configuration, where the fluorine droplet is placed too close to the polycarbonate support. This positioning increases the risk of local susceptibility-induced field inhomogeneities and may result in signal degradation.

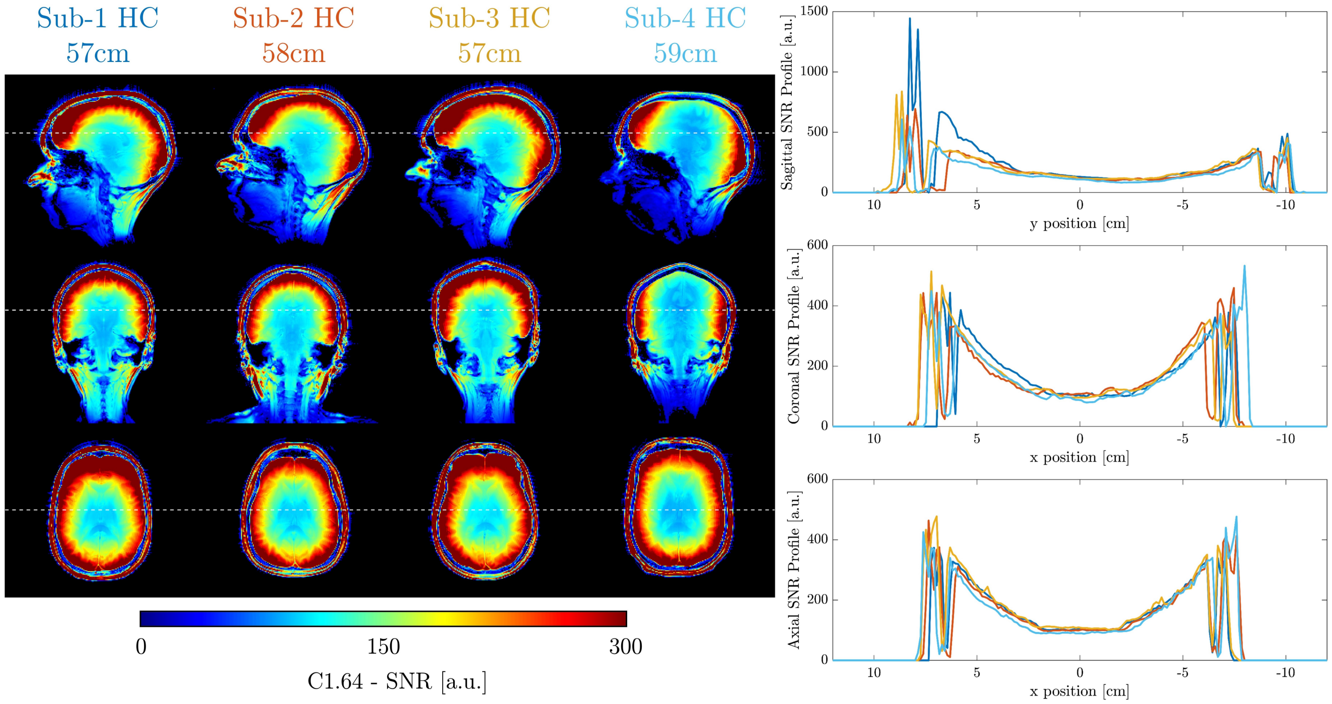

Figure S7: In vivo SNR maps and profiles obtained from the C1.64 coil. SNR maps of four subjects are displayed in sagittal, coronal, and axial views (left), with white dashed lines indicating the positions used for extracting SNR profiles (right). Head circumferences (HC) are listed above each subject for reference. The SNR profiles reveal comparable values in the central brain regions across subjects, while peripheral brain areas show inter-subject variability. Subjects with smaller head circumferences exhibit lower peripheral SNR due to increased distance from the coil helmet structure. Subject 4 demonstrates a notable reduction in SNR in the upper brain region, likely due to suboptimal head positioning.

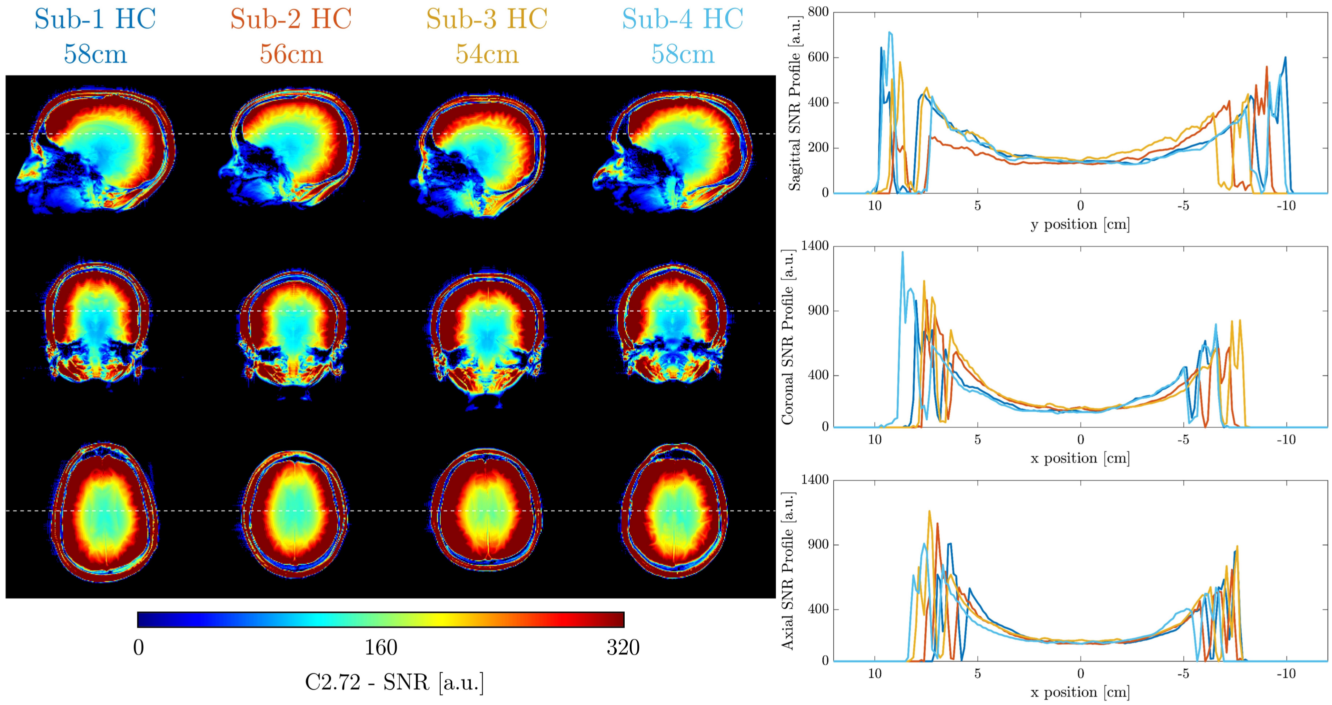

Figure S8: In vivo SNR maps and profiles obtained from the C2.72 coil. SNR maps of four subjects are displayed in sagittal, coronal, and axial views (left), with white dashed lines indicating the positions used for extracting SNR profiles (right). Head circumferences (HC) are listed above each subject. The SNR profiles reveal consistent values in the central brain regions across all subjects, with typical inter-subject variability observed in peripheral brain areas based on head sizes.

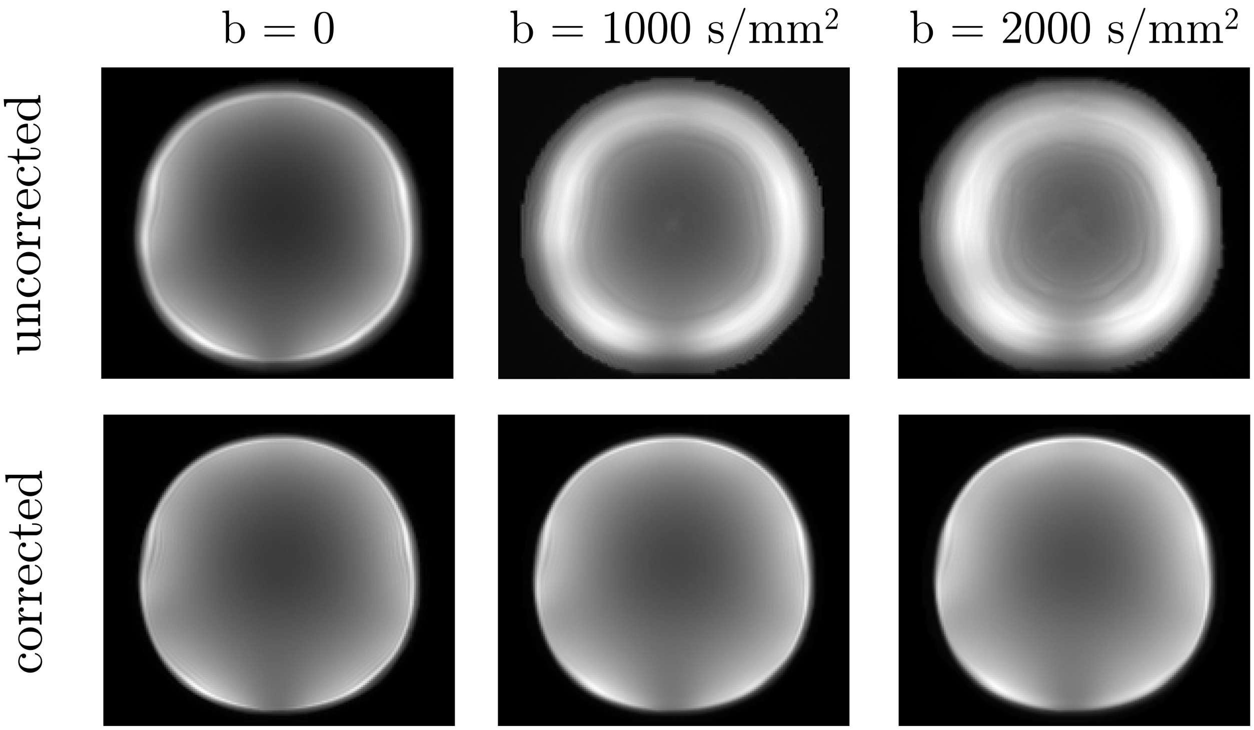

Figure S9: Field monitoring correction in low-diffusion phantom imaging on Connectome 1.0. Diffusion-weighted images of a phantom filled with low-diffusion liquid, acquired at b = 0, 1000, and 2000 s/mm^2 using the C1.64 coil on the 3T Connectome 1.0 scanner. The top row shows reconstructions using the measured trajectory of the first slice for all slices, while the bottom row applies the full set of dynamically measured 2nd-order trajectories. The dynamic correction substantially improves image sharpness, demonstrating the effectiveness of concurrent field monitoring under controlled phantom conditions.

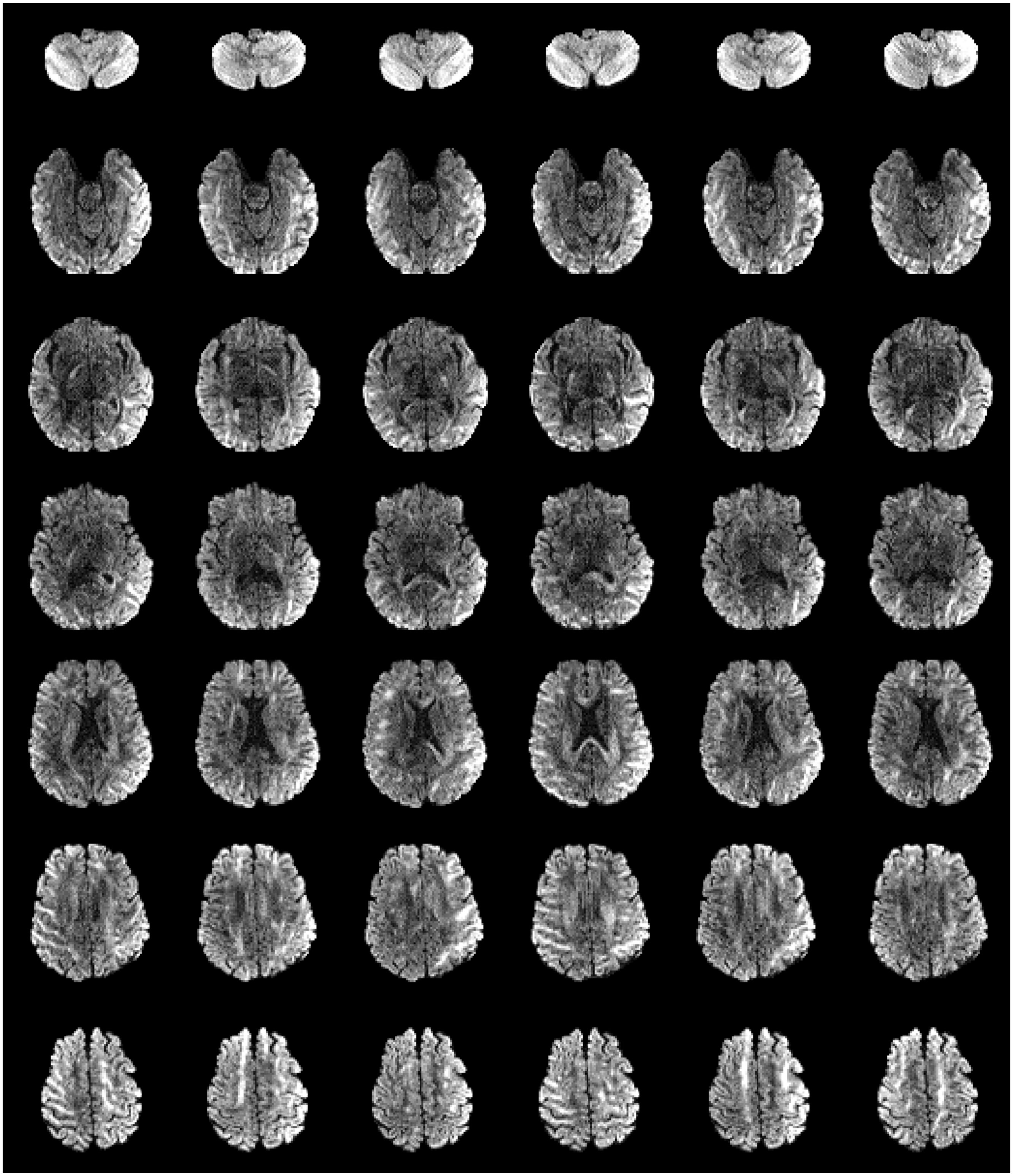

Figure S10: Full-brain diffusion-weighted images obtained from the C2.72 coil. Axial slices acquired at b = 2,500 s/mm^2 with concurrent field monitoring on the Connectome 2.0 scanner. The figure demonstrates consistent image quality across all slices, based on data acquired using six non-collinear diffusion directions.

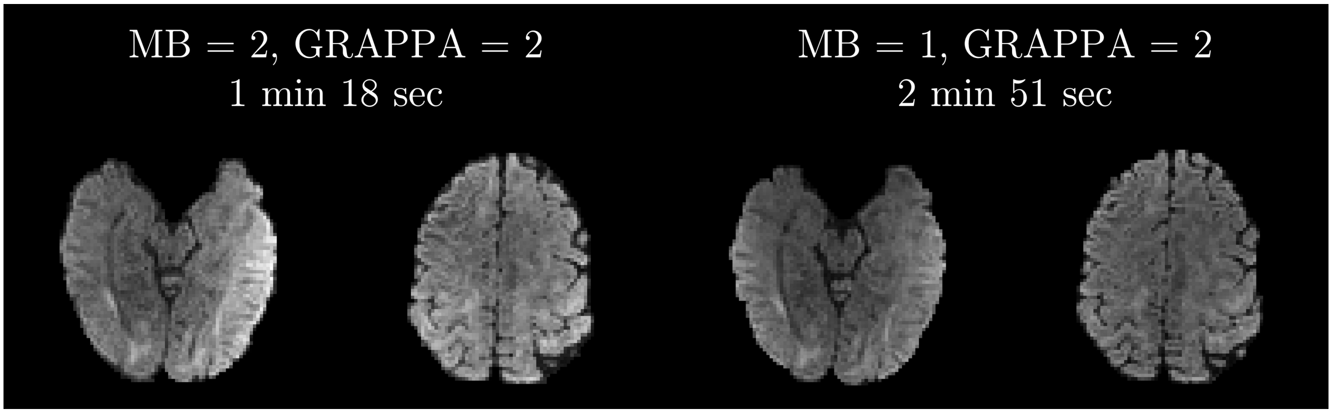

Figure S11: Comparison of diffusion-weighted images obtained with and without multiband (MB) acceleration using concurrent field monitoring image reconstruction. Two representative axial slices from a 60-slice full brain scan were acquired with the C2.72 coil at b = 2,500 s/mm^2 using different acceleration settings. The left panel shows MB = 2 and GRAPPA = 2 (scan time = 1 min 18 sec), while the right panel shows no multiband acceleration (MB = 1) and GRAPPA = 2 (scan time = 2 min 51 sec). These images demonstrate the feasibility of accelerated multi-slice in vivo diffusion imaging with field monitoring enabled by the array encoding capabilities of the C2.72 coil.

Table S1: Acquisition parameters for the DWI scans performed with the Connectome 1.0 system.

**Sequence Parameters**	**in vivo and low-diffusion phantom**
Resolution	$1.2 \times 1.2 \times 1.2$ mm^2
FoV	216×216 mm^2
Reconstructed matrix	180×180
TR	5800 ms
Phase encoding	–
No. slices	72
Bandwidth	–
Echo spacing	–
k-space trajectory	spiral
Partial Fourier	off
Parallel imaging	$R = 2$ radially undersampled trajectory repeated with 180° rotation for full effective sampling
Max. gradient strength	162 mT/m
No. directions	60 non-collinear
b_0 images	13 interspersed
b-value 1: TE, Δ, δ	1000 s/mm^2: 32 ms, 16.3 ms, 7.1 ms
b-value 2: TE, Δ, δ	2000 s/mm^2: 32 ms, 16.3 ms, 7.1 ms
Acquisition time	30 min

Table S2: Acquisition parameters for the high b-value DWI scans performed with the Connectome 2.0 system.

**Sequence Parameters**	**high b-value (C2.0 protocol)**	**high b-value (C1.0 protocol)**
Resolution	$2.0 \times 2.0 \times 2.0$ mm^3	$2.0 \times 2.0 \times 2.0$ mm^3
FoV	$230 \times 230\,mm^2$	$230 \times 230\,mm^2$
Reconstructed matrix	116×116	116×116
TR	20000 ms	20000 ms
Phase encoding	A-P	A-P
No. slices	90	90
Bandwidth	3078 Hz/pixel	3078 Hz/pixel
Echo spacing	0.37 ms	0.37 ms
k-space trajectory	cartesian	cartesian
Partial Fourier	off	off
Parallel imaging	GRAPPA $R = 2$	GRAPPA $R = 2$
Max. gradient strength	500 mT/m	500 mT/m
No. directions	6 non-collinear	6 non-collinear
b_0 images	1 at the beginning of each shell	1 at the beginning of each shell
b-value 1: TE, Δ, δ	$2500\,s/mm^2$: 33 ms, 11 ms, 8 ms	$2500\,s/mm^2$: 43 ms, 16 ms, 8 ms
b-value 2: TE, Δ, δ	$5000\,s/mm^2$: 40 ms, 15 ms, 8 ms	$5000\,s/mm^2$: 48 ms, 18 ms, 8 ms
b-value 3: TE, Δ, δ	$10000\,s/mm^2$: 40 ms, 15 ms, 8 ms	$10000\,s/mm^2$: 56 ms, 28 ms, 8 ms
b-value 4: TE, Δ, δ	$15000\,s/mm^2$: 45 ms, 20 ms, 8 ms	$15000\,s/mm^2$: 66 ms, 42 ms, 8 ms
b-value 5: TE, Δ, δ	$30000\,s/mm^2$: 57 ms, 32 ms, 8 ms	$30000\,s/mm^2$: 106 ms, 76 ms, 8 ms
Acquisition time	5 min	5 min

Table S3: Acquisition parameters for the tractography protocol performed with the Connectome 2.0 system.

**Sequence Parameters**	**tractography**
Resolution	$1.0 \times 1.0 \times 1.0$ mm^3
FoV	$210 \times 210\,mm^2$
Reconstructed matrix	210×210
TR	18000 ms
Phase encoding	A-P
No. slices	124
Bandwidth	1912 Hz/pixel
Echo spacing	0.57 ms
k-space trajectory	cartesian
Partial Fourier	6/8
Parallel imaging	GRAPPA $R = 2$
Max. gradient strength	500 mT/m
No. directions	$48^{\text{upper shell}}$ / $32^{\text{lower shell}}$ non-collinear
b_0 images	17 interspersed
b-value 1: TE, Δ, δ	$1200\,s/mm^2$: 49 ms, 13.6 ms, 3.5 ms
b-value 2: TE, Δ, δ	$2500\,s/mm^2$: 49 ms, 24 ms, 4.2 ms
Acquisition time	34 min

Table S4: Acquisition parameters for the DWI protocols with SMS acquisition performed with the Connectome 2.0 system.

**Sequence Parameters**	**MB = 2**	**no SMS**
Resolution	$2.0 \times 2.0 \times 2.0$ mm^3	$2.0 \times 2.0 \times 2.0$ mm^3
FoV	230×230 mm^2	230×230 mm^2
Reconstructed matrix	116×116	116×116
TR	7000 ms	14000 ms
Phase encoding	A-P	A-P
No. slices	60	60
Bandwidth	3448 Hz/pixel	3448 Hz/pixel
Echo spacing	0.33 ms	0.33 ms
k-space trajectory	cartesian	cartesian
Partial Fourier	off	off
Parallel imaging	GRAPPA $R = 2$	GRAPPA $R = 2$
Max. gradient strength	500 mT/m	500 mT/m
No. directions	6 non-collinear	6 non-collinear
b_0 images	2 at the beginning of each shell	2 at the beginning of each shell
b-value: TE, Δ, δ	2500 s/mm^2: 44 ms, 13.6 ms, 4.29 ms	2500 s/mm^2: 44 ms, 13.6 ms, 4.29 ms
Acquisition time	1 min 18 sec	2 min 51 sec

Table S5: Radiofrequency bench level metrics obtained from the constructed C1.64 and C2.72 head coil arrays.

**RF Bench Metrics**	**C1.64 head coil**	**C2.72 head coil**
$Q_{\mathrm{UL}}/Q_{\mathrm{L}}$–ratio	3.5	3.4
Δf shift upon loading	0.3 MHz	0.2 MHz
S_{11} matching	<-22 dB	<-18 dB
S_{21} geometrical decoupling	<-12 dB	<-12 dB
S_{21} next nearest neighbor coupling	-10 to -27 dB	-11 to -24 dB
ΔS_{21} active detuning	>37 dB	>37 dB
ΔS_{21} preamp decoupling	>18 dB	>18 dB
